# Identification of the Otopetrin Domain, a conserved domain in vertebrate otopetrins and invertebrate otopetrin-like family members

**DOI:** 10.1186/1471-2148-8-41

**Published:** 2008-02-06

**Authors:** Inna Hughes, Jonathan Binkley, Belen Hurle, Eric D Green, NISC Comparative Sequencing Program, Arend Sidow, David M Ornitz

**Affiliations:** 1 Department of Developmental Biology, Washington University School of Medicine, St. Louis, MO 63110, USA; 2 Departments of Genetics and Pathology, Stanford University Medical Center, Stanford, CA 94305, USA; 3 Genome Technology Branch, National Human Genome Research Institute, National Institutes of Health, Bethesda, MD 20892, USA; 4 NIH Intramural Sequencing Center (NISC), National Human Genome Research Institute, National Institutes of Health, Bethesda, MD 20892, USA

## Abstract

**Background:**

*Otopetrin 1 (Otop1) *encodes a multi-transmembrane domain protein with no homology to known transporters, channels, exchangers, or receptors. Otop1 is necessary for the formation of otoconia and otoliths, calcium carbonate biominerals within the inner ear of mammals and teleost fish that are required for the detection of linear acceleration and gravity. Vertebrate *Otop1 *and its paralogues *Otop2 *and *Otop3 *define a new gene family with homology to the invertebrate Domain of Unknown Function 270 genes (*DUF270*; pfam03189).

**Results:**

Multi-species comparison of the predicted primary sequences and predicted secondary structures of 62 vertebrate otopetrin, and arthropod and nematode DUF270 proteins, has established that the genes encoding these proteins constitute a single family that we renamed the Otopetrin Domain Protein (*ODP*) gene family. Signature features of ODP proteins are three "Otopetrin Domains" that are highly conserved between vertebrates, arthropods and nematodes, and a highly constrained predicted loop structure.

**Conclusion:**

Our studies suggest a refined topologic model for ODP insertion into the lipid bilayer of 12 transmembrane domains, and highlight conserved amino-acid residues that will aid in the biochemical examination of ODP family function. The high degree of sequence and structural similarity of the ODP proteins may suggest a conserved role in the intracellular trafficking of calcium and the formation of biominerals.

## Background

*Otopetrin1 *(*Otop1*) is the first described member of the *otopetrin *family, a novel gene family that encodes multi-transmembrane domain proteins. The family was named for the conserved role of *Otop1 *in the formation of otoconia and otoliths – "oto" (ear) and "petros" (stone). Otoconia are complex calcium carbonate biominerals in the utricle and saccule of the vertebrate inner ear that are required for the normal sensation of linear acceleration and gravity. Degeneration or displacement of otoconia can lead to vertigo and loss of balance [1-5]. Three mutant mice and one zebrafish model with mutations in *Otop1 *have been described: *tilted *(*tlt*) [6]; *mergulhador *(*mlh*) [7]; *inner ear defect *(*ied*) [8]; and *backstroke *(*bks*) [9], respectively. All of these mutants lack otoconia or otoliths, but have normal inner ear development. In zebrafish, the morpholino knockdown of *Otop1 *phenocopies the *tlt *mutation, showing otolith agenesis with no disruption of the patterning of the developing inner ear [9,10].

The *otopetrin *family in most vertebrates studied consists of three genes clustered in two chromosomal locations: *Otop1 *(i.e., human Chr 4p16, mouse Ch5B2) and the paralogous tandem genes *Otop2 *and *Otop3 *(i.e., human Ch17q24-25, mouse Ch11E2). Vertebrate otopetrins share a conserved gene and protein structure, with no homology to other transporters, channels, exchangers, or receptors. A preliminary secondary structure prediction based on the human, mouse, rat, zebrafish, and fugu protein sequences suggested a topology of ten transmembrane domains (TM) with cytosolic amino and carboxy termini. Additionally, tBlastn searches in the EST and genomic databases identified regions of homology with the DUF270 domain in a number of arthropod and nematode proteins. DUF270 (pfam03189) is a 404 amino-acid consensus sequence of unknown function that defines the *DUF270 *family, with members in *C. elegans *and *D. melanogaster*. The two regions of maximum homology with DUF270 found in vertebrate otopetrins correspond to putative TM domains 3–5 and 9–10, respectively, and were initially designated DUF270-I and DUF270-II [7].

Here, we report a comparison of evolutionary constraint and hydropathy profile analysis of 62 vertebrate otopetrins and arthropod and nematode DUF270 proteins, demonstrating that the genes that encode these proteins constitute a single family that we have renamed the Otopetrin Domain Protein (*ODP*) gene family. The refined topologic model of the ODP proteins includes 12 putative TM domains clustered into three "Otopetrin Domains" (OD-I, -II, and -III, respectively), with a strong degree of sequence conservation across widely divergent groups of metazoa. These regions of highest homology and evolutionary constraint, including the FYR box in the cytoplasmic tail, may represent important functional sub-domains. Biochemical studies in transfected cells show that Otop1 modulates the manner in which cells handle intracellular calcium in response to purinergic stimuli [11]. The lack of known functional domains, such as ATP-binding domains, selectivity pores, or G-protein-binding consensus sequences, suggests that either the ODP family has a novel function that significantly differs from the activities of known channels, transporters, or receptors, or that the *ODP *genes encode novel functional motifs. We hypothesize that these motifs would likely occur within the evolutionarily constrained regions, as has been shown for other well-conserved gene families [12]. The challenge remains to define the functional domains of the ODP family, with sequence and analyses reported here providing a step in that direction.

## Results and Discussion

### Comparative sequence data set

The annotation of the *Otop1*, *Otop2*, and *Otop3 *genes in the human, mouse, rat, zebrafish, and fugu genomes is described elsewhere [7]. Orthologous otopetrin sequences were generated using a targeted sequencing approach (from dog, cow, armadillo and western clawed frog) (see methods in [13,14]) or identified through tBlastn searches of available whole-genome sequences. The phylogenetic relationships of vertebrate *otopetrin *and arthropod and nematode *DUF270 *genes were deduced from a total of 62 complete or nearly complete open reading frames in 25 species (see Table 1 for a listing of the specific species and accession numbers). Fragmentary, but clearly *otopetrin*-related, sequences were also identified in urochordates (ciona), echinoderms (urchin), and cnidarians (nematostella), however were not complete enough to include in this analysis.

**Table 1:** Otopetrin Domain Protein genes

Species	Name	Gene	Symbol	Accession No.
Human	*Homo sapiens*	otopetrin 1	*OTOP1*	NM_177998
		otopetrin 2	*OTOP2*	NM_178160
		otopetrin 3	*OTOP3*	NM_178233
Chimpanzee	*Pan troglodytes*	otopetrin 1	*Otop1*	* ENSPTRT00000029625
		otopetrin 2	*Otop2*	XM_511667
Rhesus macaque	*Macaca mulatta*	otopetrin 1	*Otop1*	XM_001097009
Mouse	*Mus musculus*	otopetrin 1	*Otop1*	NM_172709
		otopetrin 2	*Otop2*	NM_172801
		otopetrin 3	*Otop3*	NM_027132
Rat	*Rattus norvegicus*	otopetrin 1	*Otop1*	NM_181433
		otopetrin 2	*Otop2*	XM_221107
		otopetrin 3	*Otop3*	XM_001081677
Cow	*Bos taurus*	otopetrin 2	*Otop2*	XM_606240, AC148430
Dog	*Canis familiaris*	otopetrin 2	*Otop2*	XM_540422, AC149469
		otopetrin 3	*Otop3*	XM_540423, AC149469
Opossum	*Monodelphis domestica*	otopetrin 2	*Otop2*	* ENSMODT00000008924
		otopetrin 3	*Otop3*	* ENSMODG00000007075
Platypus	*Ornithorhynchus anatinus*	otopetrin 3	*Otop3*	* ENSOANG00000004377
Armadillo	*Dasypus novemcinctus*	otopetrin 2	*Otop2*	AC147459
Western clawed frog	*Xenopus tropicalis*	otopetrin 1	*Otop1*	* ENSXETT00000055844
		otopetrin 2	*Otop2*	* ENSXETP00000014996
		otopetrin 3	*Otop3*	AC166187
Chicken	*Gallus gallus*	otopetrin 1	*Otop1*	* ENSGALP00000024128
		otopetrin 3	*Otop3*	XM_420115
Japanese medaka	*Oryzias latipes*	otopetrin 1	*Otop1*	* ENSORLT00000010414
Zebrafish	*Danio rerio*	otopetrin 1	*Otop1*	NM_198803
Tetraodon	*Tetraodon nigroviridis*	otopetrin 1	*Otop1*	^† ^CAAE01014674 (CAG02008)
Three-spined	*Gasterosteus aculeatus*	otopetrin 1	*Otop1*	* ENSGACT00000012102
stickleback		otopetrin 2	*Otop2*	* ENSGACT00000014538
		otopetrin 3	*Otop3*	* ENSGACT00000019137
Fugu	*Fugu rubripes*	otopetrin 1	*Otop1*	BK000652
		otopetrin 3	*Otop3*	* SINFRUT00000140311
Yellow fever	*Aedes aegypti*	otopetrin-like b1	*OTOPLb1*	^† ^CH477312 (EAT43886)
mosquito		otopetrin-like b2	*OTOPLb2*	^† ^CH477312 (EAT43887)
		otopetrin-like c	*OTOPLc*	^† ^CH477407 (EAT41549)
Fruitfly	*Drosophila melanogaster*	otopetrin-like a	*OTOPLa*	AY071510
		otopetrin-like b	*OTOPLb*	NM_164531
		otopetrin-like c	*OTOPLc*	NM_132010
Fruitfly	*Drosophila*	otopetrin-like b	*OTOPLb*	^† ^CH379061 (EAL32988)
	*pseudoobscura*	otopetrin-like c	*OTOPLc*	^† ^CH379063 (EAL32758)
Honey bee	*Apis mellifera*	otopetrin-like a	*OTOPLa*	XM_394295
		otopetrin-like c	*OTOPLc*	XM_394296
Malaria mosquito	*Anopheles gambiae*	otopetrin-like a	*OTOPLa*	XM_311233
		otopetrin-like b1	*OTOPLb1*	XM_311078
		otopetrin-like b2	*OTOPb2*	XM_311079
		otopetrin-like c	*OTOPLc*	XM_311232
Red flour beetle	*Tribolium castaneum*	otopetrin-like a	*OTOPLa*	XM_969602
		otopetrin-like b	*OTOPLb*	XM_962801
		otopetrin-like c	*OTOPLc*	XM_969568
Nematode	*Caenorhabditis*	otopetrin-like d	*OTOPLd*	^† ^CAAC01000008 (CAE58380)
	*briggsae*	otopetrin-like e	*OTOPLe*	^† ^CAAC01000008 (CAE58381)
		otopetrin-like f	*OTOPLf*	^† ^CAAC01000008 (CAE58382)
		otopetrin-like g	*OTOPLg*	^† ^CAAC01000076 (CAE69908)
		otopetrin-like h	*OTOPLh*	^† ^CAAC01000035 (CAE63792)
		otopetrin-like i	*OTOPLi*	^† ^CAAC01000052 (CAE65819)
Nematode	*Caenorhabditis*	otopetrin-like d1	*OTOPLd1*	^†† ^U64845 (AAC48028)
	*elegans*	otopetrin-like d2	*OTOPLd2*	NM_071735
		otopetrin-like e	*OTOPLe*	^†† ^U64845 (AAC48027)
		otopetrin-like f	*OTOPLf*	^†† ^U64845 (AAC48029)
		otopetrin-like g	*OTOPLg*	^††† ^AL009170 (CAA15637)
		otopetrin-like h	*OTOPLh*	^†† ^AF045639 (AAC02566)
		otopetrin-like i	*OTOPLi*	^†† ^U28737 (AAL02486)

In some instances, the nucleotide accession number corresponds to a ^†^scaffold, ^††^cosmid, or ^†††^fosmid record; in those cases, the accession number of the Otop or OTOPL annotation (protein) is indicated in parenthesis.

* ENSEMBL accession number

### Phylogenetic relationships and revised nomenclature of vertebrate otopetrins and arthropod and nematode DUF270 genes

**Figure 1:**
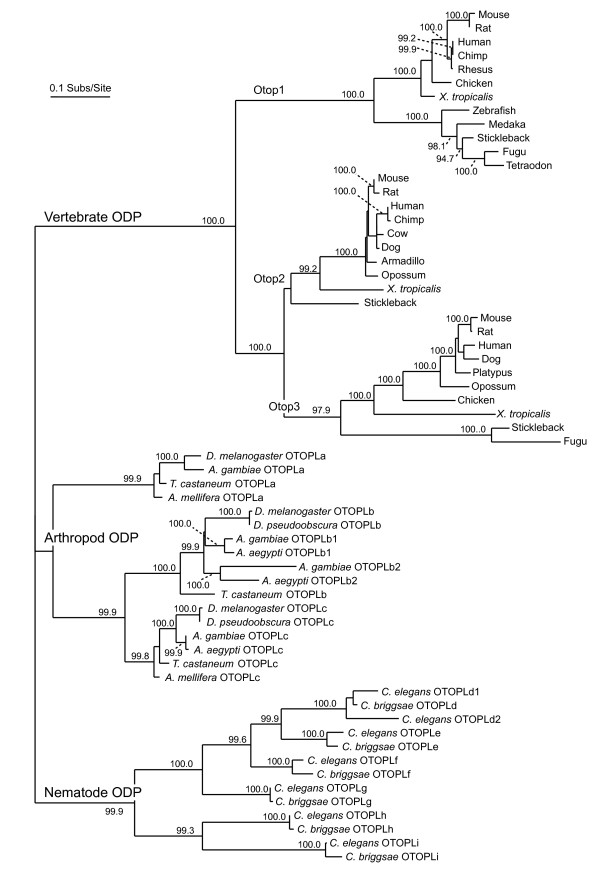
**Phylogeny of the Otopetrin Domain Protein (ODP) family**. Maximum-likelihood phylogenetic tree created from the multi-sequence alignment of 62 ODPs (see additional file 1). The vertebrate, arthropod, and nematode sequences form distinct monophyletic groups, each containing three or more paralogous groups. Some nematode and arthropod sequences appear to have undergone additional gene-duplication events, creating species-specific paralogs (designated with a 1 or 2 following the gene symbol). Branch labels are bootstrap values for 1000 replicates. Unlabeled internal branches have bootstrap values less than 90.0.

A maximum-likelihood phylogenetic tree was created from the multi-sequence alignment of each encoded protein (Figure 1). The vertebrate, arthropod, and nematode sequences form distinct monophyletic groups, each containing three or more paralogous groups. This arrangement suggests that the ancestral metazoan genome may have contained a single otopetrin-like gene, with subsequent duplications giving rise to the paralogs in the different phyla after the three lineages diverged. Based on the positions in the tree of the named mouse and human sequences, the three vertebrate paralogous groups correspond to Otop1, Otop2, and Otop3. Otop2 and Otop3 are more closely related to each other than either is to Otop1, a clustering that parallels the genomic organization of the *Otop *genes in the vertebrate genomes. The arthropod and nematode DUF270 sequences, in which encoded proteins cluster independently in the tree from the vertebrate otopetrin sequences, have been renamed as otopetrin-like proteins (OTOPL), and the paralogous groups have been assigned arbitrary letters. This is in agreement with the HUGO gene nomenclature committee guidelines for gene families and grouping [15]. Like vertebrates, arthropods also have three paralogous groups of OTOPLs. The grouping in nematodes is more complex: there appears to be three major groups of OTOPLs, as in vertebrates and arthropods, but each group itself contains two or more paralogous groups as a result of species-specific gene duplications. In summary, vertebrate *otopetrins *and arthropod and nematode *OTOPL *genes have been grouped as a single family that we named collectively the Otopetrin Domain Protein (*ODP*, see below) gene family.

### Refined topological model for ODP insertion into the lipid bi-layer

**Figure 2:**
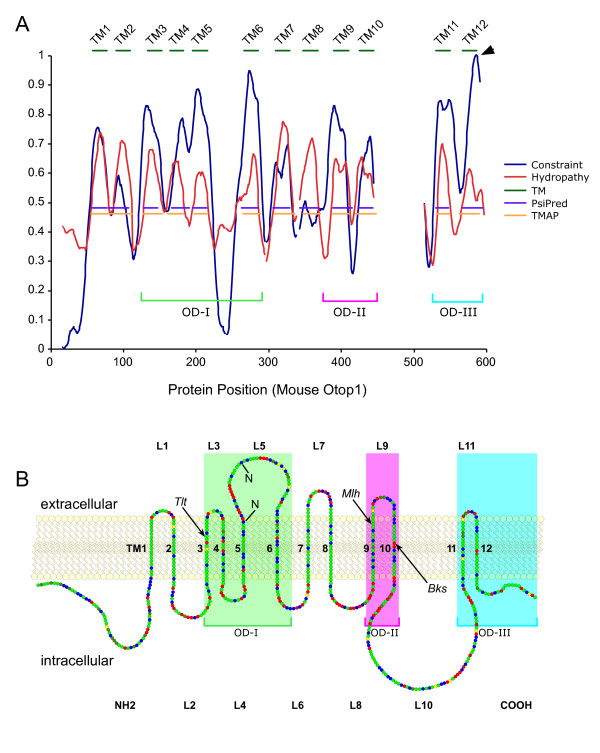
**Predicted secondary structure and topologic model for Otop1 insertion into the lipid bilayer**. A) Hydrophobicity (red) and evolutionary constraint (blue) are plotted against the amino-acid position of mouse Otop1. A total of 12 evolutionarily constrained regions are found in the ODP family that are highly hydrophobic and have a helical structure consistent with TM domains (dark green), as predicted by TMAP (orange) and PsiPred (purple). Green, pink, and blue brackets define the highly conserved subdomains: Otopetrin Domain-I, -II, and -III (OD-I, OD-II, and OD-III, respectively). B) Linear model of mouse Otop1a inserted in a lipid bilayer, in which each amino acid is represented as a circle and the chemical properties of amino-acids are denoted by color: charged residues (red), polar residues (blue), and non-polar residue (green). Cysteine (yellow) and proline (dark green) are noted. The two consensus N-glycosylation sites (N) are indicated in loop 5. The predicted intracellular and extracellular loops and TM domains are numbered L1 to L11 and TM1 to TM12, respectively. The locations of the *tlt*, *mlh*, and *bks *mutations are noted by arrows. The three OD subdomains are shaded with the color code used in A.

Conserved primary sequence is indicative of an underlying conserved tertiary structure, and the evolutionary information contained in an alignment of related sequences can be leveraged to improve predictions of shared structures [16]. We took advantage of the deep multi-sequence alignment and phylogenetic tree of the ODP family to reexamine the predicted topology of the ODPs (Figure 2A). A hydropathy profile was generated that employs phylogenetic averaging [17] on hydropathy scale values for amino acids [18] to improve the detection of conserved hydrophobic regions, which might correspond to TM domains. The hydropathy profile revealed 12 strong hydrophobic regions, ten of which overlap with the originally predicted TM domains [7]. Likewise, the MEMSAT3 [19] and TMAP [20] algorithms, which take into account leveraged evolutionary information, also predicted 12 TM helices for ODP family members that overlap well with the constrained regions and hydrophobic regions in our profile (Figure 2A).

The refined topological model for the ODP family thus consists of 12 TM domains, with both the N- and C-termini in the cytosol, and in which the two newly identified TM domains are TM4 and TM10, respectively. As shown in Figure 2B, there are three discrete regions with maximum evolutionary constraint among vertebrates, arthropods and nematodes, which we have designated Otopetrin Domain (OD) -I, -II, and -III, respectively. Among the TM domains, TM2 and TM8 show the poorest conservation and evolutionary constraint across species. On the other hand, the loops connecting the TM domains show little sequence conservation or evolutionary constraint, strongly suggesting that the TM domains are the primary functional regions of the ODP family (Figure 2A and Additional file 1). Despite the poor loop sequence conservation, the number of amino acids in 8 of the 11 loops separating TM domains is highly conserved (Table 2), suggesting that the spacing of most of the TM domains relative to one another may be important for the tertiary structure and function of ODP family members. Of note, the length of loop 5, within OD-I, is highly variable across all phyla, but conserved in vertebrates (48 ± 4 amino acid residues), as are all other loops except for loop 10.

**Table 2:** Transmembrane domain inter-loop length (amino-acids)

	NH_2 _(I)	L1 (O)	L2 (I)	L3 (O)	L4 (I)	L5 (O)	L6 (I)	L7 (O)	L8 (I)	L9 (O)	L10 (I)	L11 (O)	COOH (I)
Mouse Otop1	58^#^	12^#^	23	10	11	50	21	9	19	11	85	16	13^#^
Rat Otop1	58	12	23	10	11	50	21	9	19	11	85	16	13
Human OTOP1	61	12	23	10	11	50	21	9	19	11	95	16	13
Chimp Otop1	61	12	23	10	11	50	21	9	19	11	95	16	13
Rhesus Otop1	61	12	23	10	11	50	21	9	19	11	93	16	13
Chicken Otop1	11*	12	23	10	11	50	21	9	19	11	98	16	13
*X. tropicalis *Otop1	39	12	23	10	11	47	21	9	19	11	96	16	13
Zebrafish Otop1	54	12	23	10	11	47	22	9	19	11	75	16	13
Medaka Otop1	52	12	23	10	11	49	22	9	19	11	73	16	13
Stickleback Otop1	51	12	24	10	11	47	22	9	19	11	87	16	13
Fugu Otop1	51	12	23	10	11	47	22	9	19	11	87	16	13
Tetraodon Otop1	50	12	23	10	11	47	22	9	19	11	87	16	12

Mouse Otop2	30	12	23	10	11	54	26	9	19	11	67	16	13
Rat Otop2	30	12	23	10	11	54	26	9	19	11	67	16	13
Human OTOP2	30	12	23	10	11	53	26	9	19	11	67	16	13
Chimp Otop2	30	12	23	10	11	53	26	9	19	11	67	16	13
Dog Otop2	30	12	23	10	11	53	26	9	19	11	67	16	13
Cow Otop2	30	12	23	10	11	53	26	9	19	11	67	16	13
Armadillo Otop2	30	12	23	10	11	53	26	9	19	11	67	16	13
Opossum Otop2	30	12	23	10	11	46	32	6	19	11	65	16	13
*X. tropicalis *Otop2	31*	12	22	10	11	54	25	8	19	11	62	16	13
Stickleback Otop2	31*	12	23	10	11	52	20	12	19	11	64	16	13

Mouse Otop3	70	12	23	10	11	44	24	8	19	11	54	16	13
Rat Otop3	71	12	23	10	11	44	24	8	19	11	54	16	13
Human OTOP3	89	12	23	10	11	44	24	8	19	11	54	16	13
Dog Otop3	71	12	23	10	11	44	24	8	19	11	54	16	13
Opossum Otop3	59*	12	23	10	11	44	24	8	19	11	58	16	13
Platypus Otop3	51*	12	23	10	11	44	24	8	19	11	51	16	13
Chicken Otop3	24	12	23	10	11	44	23	8	19	11	62	16	13
*X. tropicalis *Otop3	34	12	23	10	11	43	22	8	19	11	46	16	13
Stickleback Otop3	48	12	23	10	11	45	28	8	19	11	60	16	13
Fugu Otop3	1*	12	23	10	11	45	27	8	19	11	44	16	11

*D. melanogaster *OTOPLa	64	14	79	13	11	337	43	8	15	9	14	16	19
*A. gambiae *OTOPLa	65	14	69	13	11	264	43	8	15	9	14	16	19
A. mellifera. OTOPLa	73	14	74	13	11	202	44	8	15	9	14	16	19

*T. castaneum *OTOPLa	65	14	79	13	11	148	42	8	15	9	14	16	19
*D. melanogaster *OTOPLb	76	14	26	13	12	94	34	8	20	10	14	16	19
*D. pseudoobscura *OTOPLb	76	14	26	13	12	94	34	8	20	10	14	16	19
*A. gambiae *OTOPLb1	26	14	28	13	12	91	37	8	21	10	14	16	22
*A. aegypti *OTOPLb1	197	14	26	13	12	87	37	8	21	10	14	16	22
*T. castaneum *OTOPLb	106	14	34	14	12	43	35	13	21	7	14	16	31
*A. gambiae *OTOPLb2	163	14	32	13	12	60	41	8	19	7	14	16	21
*A. aegypti *OTOPLb2	157	14	29	13	13	75	35	8	18	10	14	16	21
*D. melanogaster *OTOPLc	0*	14	60	13	12	73	34	8	15	7	14	16	115
*D. pseudoobscura *OTOPLc	0*	14	59	13	12	74	34	8	15	7	14	16	90
*A. gambiae *OTOPLc	7*	14	70	13	12	70	33	8	15	7	14	16	21
*A. aegypti *OTOPLc	10	14	68	13	12	70	33	8	15	7	14	16	21
*A. mellifera *OTOPLc	90	14	46	13	12	74	37	8	15	7	14	16	21
*T. castaneum *OTOPLc	177	14	50	13	12	79	33	8	15	7	14	16	21
*C. elegans *OTOPLd1	38	14	31	9	11	59	25	7	16	10	14	13	36
*C. briggsae *OTOPLd	47	14	34	9	11	59	25	7	16	10	14	13	37
*C. elegans *OTOPLd2	55	14	34	9	11	59	25	7	16	10	12	13	38
*C. elegans *OTOPLe	50	14	36	9	11	69	22	7	16	11	14	13	34
*C. briggsae *OTOPLe	42	14	36	9	11	67	22	7	16	11	14	13	37
*C. elegans *OTOPLf	65	14	35	9	11	93	25	7	16	10	14	13	38
*C. briggsae *OTOPLf	71	14	36	9	11	99	25	7	16	10	14	13	48
*C. elegans *OTOPLg	64	13	34	9	11	72	26	7	16	11	14	13	53
*C. briggsae *OTOPLg	64	13	34	9	11	73	26	7	16	11	14	13	55
*C. elegans *OTOPLh	56	17	81	9	11	75	25	7	22	11	13	16	34
*C. briggsae *OTOPLh	66	17	47	9	11	70	25	7	22	11	13	16	34
*C. elegans *OTOPLi	51	18	29	14	11	46	27	5	22	10	16	16	30
*C. briggsae *OTOPLi	51	18	30	14	11	48	27	5	22	10	16	16	30

Average	62.4	13.2	33.7	10.8	11.2	70.5	27.6	8.1	18.2	10.2	43.1	15.6	23.2

SD	37.3	1.5	17.0	1.6	0.5	50.2	6.5	1.2	2.0	1.4	30.6	1.1	18.3

^#^Number of amino-acid residues within the N-terminal, interloop, and C-terminal domains. **(I)**, inner loop; **(O)**, outer loop; SD, standard deviation.*Incomplete N-terminal sequence data were excluded from N-terminal loop length calculations.

### Homology between Otop and OTOPL sequences extends beyond the canonical DUF270 domain

**Figure 3:**
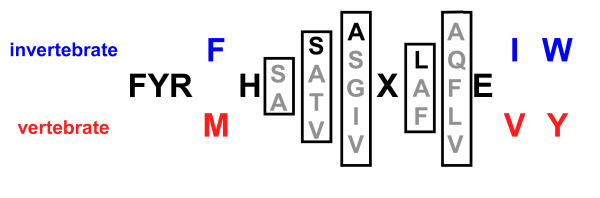
**FYR box consensus sequence for the ODP family C-terminal tail**. Residues in bold are shared by all ODP family members, X is any hydrophobic amino acid, blue residues are specifically conserved in arthropod and nematode members, and red amino acids are conserved among vertebrate members. Grey, bracketed residues represent common variants at each less-conserved position. The dark residue within each bracket represents the most common amino-acid variant at that position, if one can be identified.

DUF270 (pfam03189) is a 404 amino-acid consensus sequence of unknown function. Early tBlastn-based database searches identified regions of homology with the DUF270 domain in both vertebrate Otop and arthropod and nematode OTOPL proteins [7], now grouped together as the ODP family. Inspection of the multi-species ODP sequence alignment suggests that the homology among ODP proteins extends beyond the canonical DUF270 domain (see Additional file 1). Specifically, the N-terminal end of the DUF270 consensus sequence can be extended to include three amino acids (**HAG**, amino acids 125–127 in mouse Otop1) that are conserved in most vertebrate (**HAG**) and nematode (**GAG**) ODPs examined. At the C-terminal end, the amino-acid conservation continues well beyond the DUF270 motif to include the entire C-terminal tail of vertebrate Otop (amino acids 584–600 in mouse Otop1). A 14-amino-acid consensus sequence for this highly conserved C-terminal tail, which we named the FYR box, is shown in Figure 3. The FYR box is a signature unique to the ODP family, and is present in all ODP proteins but not in any non-ODP sequences in the databases of ESTs and non-redundant sequences.

## Conclusion

Comparative analyses of vertebrate otopetrins and arthropod and nematode OTOPL proteins revealed that they all share a TM domain structure and significant conservation of amino-acid sequence, suggesting that they constitute a single protein family, here renamed the ODP family. We have expanded the domains of homology to more accurately reflect the extent of sequence conservation between vertebrates, arthropods and nematodes, and have identified three evolutionarily constrained TM domain-rich areas that we have designated as Otopetrin Domains.

OD-I and OD-III are the most highly conserved regions of the ODP family. *Tlt *mice carry a missense mutation (Ala_151_→Glu), which alters the hydrophobicity of the predicted TM3 domain within OD-I, and leads to a presumed alteration in the membrane insertion or activity of Otop1 and otoconial agenesis [7]. The OD-II evolutionarily constrained region was not identified in the initial modeling, but mutations in Otop1 within this conserved segment of the protein have been shown to cause otolith/otoconial agenesis in *bks *mutant fish (Glu_429_→Val) [9] and in *mlh *mutant mice (Leu_408_→Gln) [7] (Figure 2B), suggesting that this region is functionally important.

Initial modeling of the OTOP proteins suggested a 10 TM domain model with cytosolic N- and C-termini [7]. This model had several problems, including that sites consistent with the consensus sequence for N-glycosylation were predicted to be cytosolic. The 12 TM domain model predicted by hydrophobicity and evolutionary constraint analysis places the proposed glycosylation sites in the extracellular space (Figure 2B), and suggests that it may reflect a more accurate version of OTOP insertion into the lipid bilayer. Interestingly, the missense mutations in the *tlt*, *mlh*, and *bks *animal models, which lead to functional loss of OTOP1 activity, each occur within highly conserved transmembrane domains; such mutations often alter the hydrophobicity of the conserved TM domain, which may lead to alterations in the ability of the protein to insert and orient in membranes.

*Otop1 *is required for the formation of vertebrate otoconia, a process that involves calcium carbonate biomineralization and requires the regulation of intracellular calcium. Biochemical studies in transfected cells show that OTOP1 modulates the manner in which cells handle intracellular calcium in response to purinergic stimuli [11]. The mechanisms of calcium carbonate biomineralization are highly conserved in the development of otoconia and otoliths in the vertebrate inner ear, the formation of the avian eggshell, the mineralization of the arthropod exoskeleton, and the development of other mineralized structures such as the mollusk shell [21-23]. There is evidence that some *ODP *family members are expressed in tissues associated with calcium secretion and calcium carbonate-based mineralization. In particular, ESTs from *Callinectes sapidus *(Blue crab) reveal strong expression of the *D. melanogaster OTOPLb *ortholog in hypodermal tissues that are required for calcium mobilization during the mineralization of the chitinous exoskeleton [24]. *ODP *mRNAs are also expressed in the hemocytes of various invertebrate species, which have been associated with the development of mineralized structures in mollusks [25]. In mammals, *Otop1 *is expressed in the lactating mammary gland [7], perhaps functioning in the secretion of calcium into milk. Taken together, the sequence homology, structural constraint, and expression pattern suggest a conserved role for members of the *ODP *family in the formation of mineralized structures. Further examination of ODPs and continued characterization of natural and induced mutations in these proteins through both physiologic and topologic studies may assist in better understanding the mechanisms of establishing and maintaining mineralized structures throughout the animal kingdom.

## Methods

### Sequence collection

Orthologous *Otopetrin *sequences were generated by a targeted sequencing approach, or identified through tBlastn searches of available whole-genome sequences. For the targeted sequencing, BAC clones were isolated from the following libraries maintained by the BACPAC Resources Center [14,26,27]: dog (*Canis familiaris*; RPCI-81), cow (*Bos Taurus*; CHORI-240), armadillo (*Dasypus novemcinctus*; VMRC-5), and western clawed frog (*Xenopus tropicalis*; CHORI-216). Specifically, each library was screened using pooled sets of oligonucleotide-based probes designed from the established sequence of the mouse *Otop1 *or *Otop2/Otop3 *subloci (on mouse Ch5B2 and Ch11E2, respectively). After isolation and mapping, a total of four BACs (accession numbers AC148430, AC149469, AC147459, and AC166187) were shotgun sequenced and subjected to sequence finishing, as described [28]. The complete gene structures were determined based on alignments to mouse RefSeq mRNAs or species-specific mRNA, when available. For the tBlastn searches, we used mouse *Otop1*, *-2*, and *-3 *to query vertebrate genome sequences, and *Drosophila OTPLa*, *-b*, and *-c *and *C. elegans OTOPLd1, -e, -f, -g, -h*, and -*i *to query arthropod and nematode genome sequences (see Table 1 for sequence accession numbers).

### Alignment, phylogenic tree generation, and evolutionary constraint versus hydropathy analysis

The initial protein sequence alignment was performed with ProbCons [29], and a preliminary phylogenetic tree was built with SEMPHY [30] using only the most confidently aligned regions of the multi-sequence alignment. The sequences were then divided into smaller groups based upon their relatedness according to the tree. Each group was re-aligned with Probcons, and each of these sub-alignments was manually adjusted. ClustalW [31,32] was then used to profile-align these sub-alignments, producing the final, full alignment. The final phylogenetic tree was constructed using SEMPHY, constraining the topology to conform to SEMPHY trees built from the sub-alignments. 1000 bootstrap replicates were generated for each subtree as well as the final tree. The bootstrap values shown in Figure 1 are from the lowest-level tree in which the given branch occurs.

Evolutionarily constrained regions were detected essentially as described previously [12]. The final alignment and tree were used to calculate single-site evolutionary rates with the empirical Bayesian version of the program Rate4Site [33]. These single-site rate values were smoothed using sliding-windows of weighted averaging. In each 17-position-wide window, the value at the center position of the window was given the highest relative weight, and the relative weight decreased linearly for the values on either side to the edge of the window. The resulting weighted average was assigned to the position in the protein corresponding to the center of the window. To produce the evolutionary constraint profile, the rates were then converted to relative constraint by normalizing to a range between 0 and 1, inverted by subtracting from 1 (because a region of low evolutionary rate is under high evolutionary constraint), and plotted against the position in the protein.

To produce the hydropathy profile, the hydropathy-scale value [18] for each amino acid in a column of the multi-sequence alignment (corresponding to a single position on the profile) was multiplied by a weighting factor that reflects the fractional contribution of the corresponding sequence to the total sequence diversity represented [17]. The hydropathy score at each position is the sum of these values. These single-position values were smoothed using the same sliding-windows weighted averaging scheme applied to the rate values above, normalized to vary between 0 and 1, and plotted against the position in the protein.

## Authors' contributions

IH carried out the analysis and drafted the manuscript. JB carried out the analysis and drafted the manuscript. BH carried out the analysis and drafted the manuscript. EDG edited the manuscript. NISC Comparative Sequencing Program provided sequence data. AS edited the manuscript. DMO carried out the analysis and drafted the manuscript. All authors read and approved the final manuscript.

## Supplementary Material

Additional File 1 **CLUSTALW alignment of known and predicted ODP family members**. The sources of the protein sequences utilized in this alignment are listed in Table 1. Predicted TM domains are shaded (tan) and numbered TM1 to TM12. Inter-TM loops are numbered L1 to L11. Otopetrin Domains OD-I, -II, and -III are shaded in green, purple, and blue, respectively. Dashes indicate sequence gaps. Click here for file
