# Supplementary material for: Identification of the Otopetrin Domain, a conserved domain in vertebrate otopetrins and invertebrate otopetrin-like family members
Source: BMC Evol Biol. 2008 Feb 6;8:41. doi: 10.1186/1471-2148-8-41 (PMC2268672; doi:10.1186/1471-2148-8-41)
Supplement: Additional File 1 — CLUSTALW alignment of known and predicted ODP family members. The sources of the protein sequences utilized in this alignment are listed in Table 1. Predicted TM domains are shaded (tan) and numbered TM1 to TM12. Inter-TM loops are numbered L1 to L11. Otopetrin Domains OD-I, -II, and -III are shaded in green, purple, and blue, respectively. Dashes indicate sequence gaps. [file 1471-2148-8-41-S1.PDF]

NH$_2$

```
Mouse Otop1                 ---------------------------------------------------------------------------------------------------------------------------------------------MPGGPGAPSSPAASSGSSRAAP
Rat Otop1                   ---------------------------------------------------------------------------------------------------------------------------------------------MPGDRGALSSPAASSGSPSAAP
Human OTOP1                 ---------------------------------------------------------------------------------------------------------------------------------------------MLEGLGSPASPRAAASASVAGS
Chimp Otop1                 ---------------------------------------------------------------------------------------------------------------------------------------------MLEGLGSPASPRAAASASVAGS
Rhesus Otop1                ---------------------------------------------------------------------------------------------------------------------------------------------MLEGLGSPASPRAAASTSVVGS
Chicken Otop1               -------------------------------------------------------------------------------------------------------------------------------------------------------------------
X. tropicalis Otop1         -------------------------------------------------------------------------------------------------------------------------------------------------------------------
Zebrafish Otop1             ----------------------------------------------------------------------------------------------------------------------------------------------------MVEHGGTDSMWLNKY
Medaka Otop1                ---------------------------------------------------------------------------------------------------------------------------------------------------RMEGDGGLDVSCLNKY
Stickleback Otop1           ---------------------------------------------------------------------------------------------------------------------------------------------------TMVEHSGLDIMCLNKY
Fugu Otop1                  ---------------------------------------------------------------------------------------------------------------------------------------------------MMVEHNGLDIMCSNKY
Tetraodon Otop1             ----------------------------------------------------------------------------------------------------------------------------------------------------MVEPNGLGVLCLSKY
Mouse Otop2                 -------------------------------------------------------------------------------------------------------------------------------------------------------------------
Rat Otop2                   -------------------------------------------------------------------------------------------------------------------------------------------------------------------
Human OTOP2                 -------------------------------------------------------------------------------------------------------------------------------------------------------------------
Chimp Otop2                 -------------------------------------------------------------------------------------------------------------------------------------------------------------------
Dog Otop2                   -------------------------------------------------------------------------------------------------------------------------------------------------------------------
Cow Otop2                   -------------------------------------------------------------------------------------------------------------------------------------------------------------------
Armadillo Otop2             -------------------------------------------------------------------------------------------------------------------------------------------------------------------
Opossum Otop2               -------------------------------------------------------------------------------------------------------------------------------------------------------------------
X. tropicalis Otop2         -------------------------------------------------------------------------------------------------------------------------------------------------------------------
Stickleback Otop2           -------------------------------------------------------------------------------------------------------------------------------------------------------------------
Mouse Otop3                 -----------------------------------------------------------------------------------------------------------------------------------MASQTSAPAEPAPMPSPEAKTTEGAS-SYDQA
Rat Otop3                   -----------------------------------------------------------------------------------------------------------------------------------MASQTSAPEPAPMPSSEAETTEAASTYDQANM
Human OTOP3                 -----------------------------------------------------------------------------------------------------------------MGRGARAAAAQSRWGRASRASVSPGRTIRSAPAVGEAQETEAAPEKENRV
Dog Otop3                   -----------------------------------------------------------------------------------------------------------------------------------MPAQASAPSQAPSTASPEAQETGAAPAGENQV
Opossum Otop3               -----------------------------------------------------------------------------------------------------------------------------------------------SSTTPSLPSPDPQGIPETTA
Platypus Otop3              -------------------------------------------------------------------------------------------------------------------------------------------------------ETAAAPGKENGV
Chicken Otop3               -------------------------------------------------------------------------------------------------------------------------------------------------------------------
X. tropicalis Otop3         -------------------------------------------------------------------------------------------------------------------------------------------------------------------
Stickleback Otop3           ---------------------------------------------------------------------------------------------------------------------------------------------------------MNADPGATEA
Fugu Otop3                  -------------------------------------------------------------------------------------------------------------------------------------------------------------------
D. melanogaster OTOPLa      ---------------------------------------------------------------------------------------------------------------------------------MGGGEVKVATVDVEGG--DNMATLPVSR------
A. gambiae OTOPLa           --------------------------------------------------------------------------------------------------------------------------------MVGGGEMKVATVDVESN--DNMATLPVSR------
A. mellifera. OTOPLa        --------------------------------------------------------------------------------------------------------------------------------MVGGGDCKVATVEVEAAAADNTATLPVTRPLNPQN
T. castaneum OTOPLa         --------------------------------------------------------------------------------------------------------------------------------MVGGGELKVATVEVESV--DNMATLPVSR------
D. melanogaster OTOPLb      ----------------------------------------------------------------------------------------------------------------------------MVDRNSGVQYERMPTVTFSNLQGTASGSTATINFQSGAT
D. pseudoobscura OTOPLb     ----------------------------------------------------------------------------------------------------------------------------MVERNSAIEYQRMPTVTFSNLQSTASGSTATINLQSGTA
A. gambiae OTOPLb1          -------------------------------------------------------------------------------------------------------------------------------------------------------------------
A. aegypti OTOPLb1          MHHASTILWMSYQEEAENPVLPTRNWAGKCEQVDIDSDDRSALKEKQQQQLLLVPKSDGEPIQLIPKAATFATMPVKYNHHGSTSLLDQIPEDSHPEKKPASTSHTN---GSTPRRPSIVVQDILSTHRPSGIMSALRRGSMAWLPGRSKSTHHGQDNESNMG
T. castaneum OTOPLb         ----------------------------------------------------------------------------------------------MDTVHEENSPAPQVPQSSLAMETLDEEDASRSSSRRPSAIIAALRRPSQAIALSAAHAVMNQRRYLLGL
A. gambiae OTOPLb2          ---------MASSPPASFRTVRTETGHSRS-----------------LSELDVGARRNVENVHPPPDEREPAAARVSARDNSAASSARPSPHQSRRGTLDSVELVERYHFAKNPNRSGSHSPSLYESRRRLSSFGAQKRAFLELIRKQGSRLSLARKFSEV--
A. aegypti OTOPLb2          ---------MDEFEWVAGVATVDESGRRKTERERLNTVTEESASESNMTQLAQIKTPLMEPIQTSP------------KLTPNVSFA--YQPGSRRHTGHSTDLEDQ----PSPDAGGSRRPSLFNALPRM-SFSSHKKHLIEFLQRHGSKLSLNHKDDSK--
D. melanogaster OTOPLc      -------------------------------------------------------------------------------------------------------------------------------------------------------------------
D. pseudoobscura OTOPLc     -------------------------------------------------------------------------------------------------------------------------------------------------------------------
A. gambiae OTOPLc          -------------------------------------------------------------------------------------------------------------------------------------------------------------------
A. aegypti OTOPLc          -------------------------------------------------------------------------------------------------------------------------------------------------------------------
A. mellifera. OTOPLc       --------------------------------------------------------------------------------------MRKHGLFSWPGLP-----------------VALPSPASPSAVIVSPETLSRHSSSSPSRAHRPPQFALALPCSSQPI
T. castaneum OTOPLc        --------MDDSSPDLSLKLRRGSSDSRDSFYMDFDKGIDSDIEEMATTSAADLGDTTADTT-----------------ATPTALGYGKTEGESWPTIPPQNMSGGSRSSALGGGDPLLPSPASPSAVIVSPETLSHHSSPSKSTKQTAHVQIAPPPKTLSPG
C. elegans OTOPLd1         -------------------------------------------------------------------------------------------------------------------------------------------------------------------
C. briggsae OTOPLd         ----------------------------------------------------------------------------------------------------------------------------------------------------------MSLNESLKN
C. elegans OTOPLd2         ---------------------------------------------------------------------------------------------------------------------------------------------------MTNIVVRRDSQTLEAS
C. elegans OTOPLe          --------------------------------------------------------------------------------------------------------------------------------------------------------MADKERLRLKT
C. briggsae OTOPLe         ----------------------------------------------------------------------------------------------------------------------------------------------------------------MKT
C. elegans OTOPLf          --------------------------------------------------------------------------------------------------------------------------------------MDNNYNSDVHEPLREKESIVSVASTSTAP
C. briggsae OTOPLf         ----------------------------------------------------------------------------------------------------------------------------------MSWRERKNPNSMLLEPLREKDSIVSVASTSTAP
C. elegans OTOPLg          -----------------------------------------------------------------------------------------------------------------------------------------MVDEQLKIGATPMYSPSHNPIVIADD
C. briggsae OTOPLg         -----------------------------------------------------------------------------------------------------------------------------------------MVDEQLKIGPVSLYSPGPNPIVIADD
C. elegans OTOPLh          ---------------------------------------------------------------------------------------------------------------------------------------MSFQQDIHLTTRRNPVKAEAASPMGRSL
C. briggsae OTOPLh         ------------------------------------------------------------------------------------------------------------------------------------MPHFDGLFSVVIETRRYPVKA---SPPSRSE
C. elegans OTOPLi          ------------------------------------------------------------------------------------------------------------------------------------------------------MSKATEEFDLIDR
C. briggsae OTOPLi         ------------------------------------------------------------------------------------------------------------------------------------------------------MNKEKEEFDLIDR
```

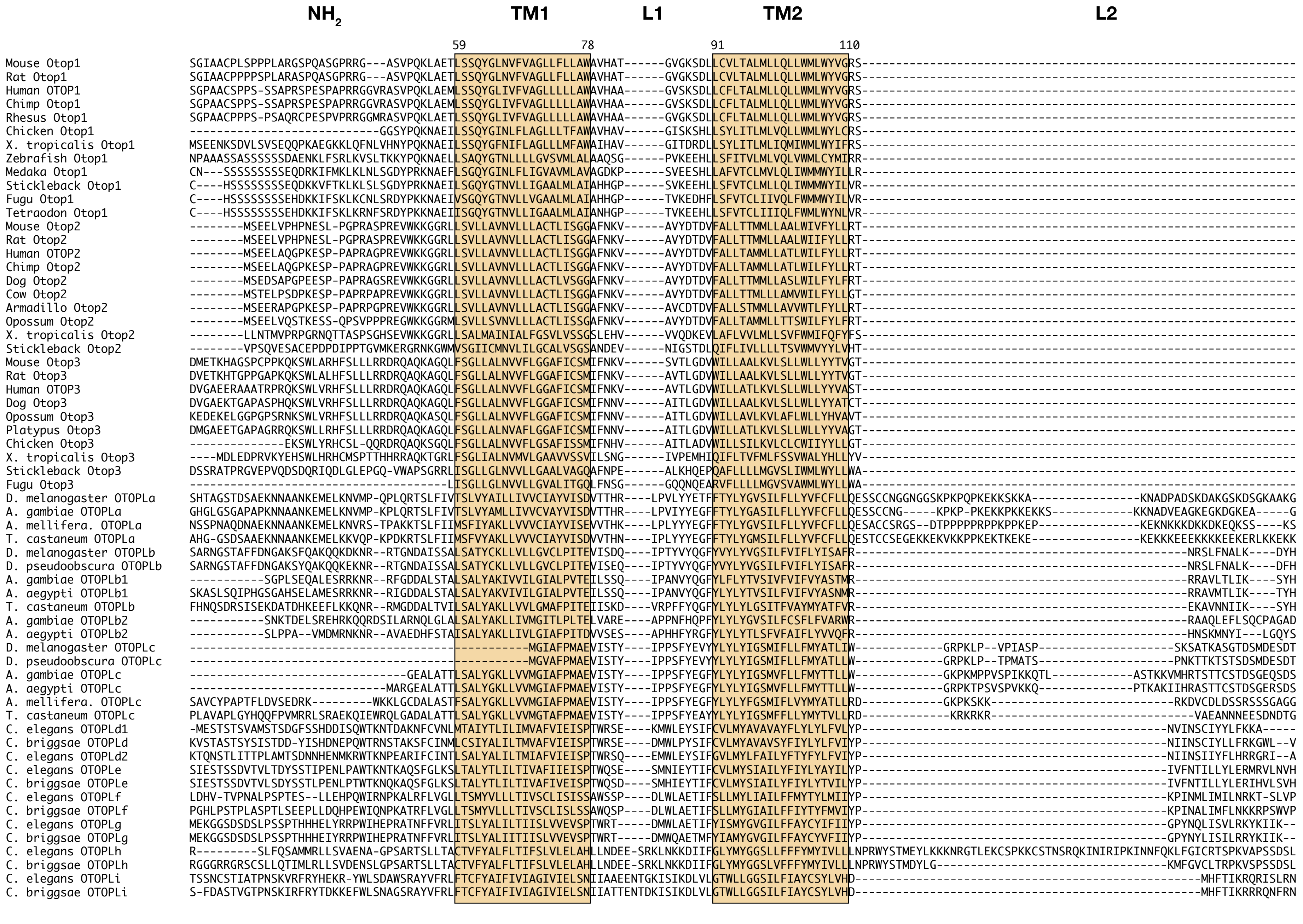
NH$_2$
TM1
L1
TM2
L2
59
78
91
110
Mouse Otop1 SGIAACPLSPPPLARGSPQASGPRRG---ASVPQKLAETLSSQYGLNVFVAGLLFLLAWAVHAT------GVGKSDLLCVLTALMLLQLLWMLWYVGRS--
Rat Otop1 SGIAACPPPPSPLARASPQASGPRRG---ASVPQKLAETLSSQYGLNVFVAGLLFLLAWAVHAT------GVGKSDLLCVLTALMLLQLLWMLWYVGRS--
Human OTOP1 SGPAACSPPS-SSAPRSPESPAPRRGGVRASVPQKLAEMLSSQYGLIVFVAGLLLLLAWAVHAA------GVSKSDLLCFLTALMLLQLLWMLWYVGRS--
Chimp Otop1 SGPAACSPPS-SSAPRSPESPAPRRGGVRASVPQKLAEMLSSQYGLIVFVAGLLLLLAWAVHAA------GVSKSDLLCFLTALMLLQLLWMLWYVGRS--
Rhesus Otop1 SGPAACPPPS-PSAQRCPESPVPRRGGMRASVPQKLAETLSSQYGLIVFVAGLLLLLAWAVHAA------GVGKSDLLCFLTALMLLQLLWMLWYVGRS--
Chicken Otop1 ----------------------------GGSYPQKNAEILSSQYGINLFLAGLLLTFAWAVHAV------GISKSHLLSYLITLMLVQLLWMLWYLCRS--
X. tropicalis Otop1 MSEENKSDVLSVSEQQPKAEGKKLQFNLVHNYPQKNAEILSSQYGFNIFLAGLLLMFAWAIHAV------GITDRDLLSYLITLMLIQMIWMLWYIFRS--
Zebrafish Otop1 NPAAASSASSSSSSDAENKLFSRLKVSLTKKYPQKNAELLSAQYGTNLLLLGVSVMLALAAQSG------PVKEEHLLSFITVLMLVQLVWMLCYMIRR--
Medaka Otop1 CN---SSSSSSSSSEQDRKIFMKLKLNLSGDYPRKNAEFLSGQYGINLFLIGVAVMLAVAGDKP------SVEESHLLAFVTCLMVLQLIWMMWYILLR--
Stickleback Otop1 C----HSSSSSSSSEQDKKVFTKLKLSLSGDYPRKNAEILSGQYGTNVLLIGAALMLAIAHHGP------SVKEEHLLSFVTCLMLLQLIWMMWYILVR--
Fugu Otop1 C----HSSSSSSSSEHDKKIFSKLKCNLSRDYPKKNAEIVSGQYGTNVLLVGAALMLAIAHHGP------TVKEDHFLSFVTCLIIVQLFWMMWYILVR--
Tetraodon Otop1 C----HSSSSSSSSEHDKKIFSKLKRNFSRDYPKKNAEIISGQYGTNVLLIGAALMLAIANHGP------TVKEEHLLSFVTCLIIIQLFWMLWYNLVR--
Mouse Otop2 --------MSEELVPHPNESL-PGPRASPREVWKKGGRLLSVLLAVNVLLLACTLISGGAFNKV------AVYDTDVFALLTTMMLLAALWIVFYLLRT--
Rat Otop2 --------MSEELVPHPNESL-PGPRASPREVWKKGGRLLSVLLAVNVLLLACTLISGGAFNKV------AVYDTDVFALLTTMMLLAALWIIFYLLRT--
Human OTOP2 --------MSEELAQGPKESP-PAPRAGPREVWKKGGRLLSVLLAVNVLLLACTLISGGAFNKV------AVYDTDVFALLTAMMLLATLWILFYLLRT--
Chimp Otop2 --------MSEELAQGPKESP-PAPRAGPREVWKKGGRLLSVLLAVNVLLLACTLISGGAFNKV------AVYDTDVFALLTAMMLLATLWILFYLLRT--
Dog Otop2 --------MSEDSAPGPEESP-PAPRAGSREVWKKGGRLLSVLLAVNVLLLACTLVSGGAFNKV------AVYDTDVFALLTTMMLLASLWILFYLFRT--
Cow Otop2 --------MSTELPSDPKESP-PAPRPAPREVWKKGGRLLSVLLAVNVLLLACTLISGGAFNKV------AVYDTDVFALLTTMLLLAMVWILFYLLGT--
Armadillo Otop2 --------MSEERAPGPKESP-PAPRPGPREVWKKGGRLLSVLLAVNVLLLACTLISGGAFNKV------AVCDTDVFALLSTMMLLAVVWTLFYLLRT--
Opossum Otop2 --------MSEELVQSTKESS-QPSVPPPREGWKKGGRMLSVLLSVNVLLLACTLISSGAFNKV------AVYDTDVFALLTAMMLLTTSWILFYLFRT--
X. tropicalis Otop2 --------LLNTMVPRPGRNQTTASPSGHSEVWKKGGRLLSALMAINIALFGSVLVSSGSLEHV------VVQDKEVLAFLVVLMLLSVFWMIFQFYFS--
Stickleback Otop2 --------VPSQVESACEPDPDIPPTGVMKERGRNKGWMVSGIICMNVLILGCALVSGSANDEV------NIGSTDLQIFLIVLLLLTSVWMVYYLVHT--
Mouse Otop3 DMETKHAGSPCPPKQKSWLARHFSLLLRRDRQAQKAGQLFSGLLALNVVFLGGAFICSMIFNKV------SVTLGDVWILLAALKVLSLLWLLYYTVGT--
Rat Otop3 DVETKHTGPPGAPKQKSWLALHFSLLLRRDRQAQKAGQLFSGLLALNVVFLGGAFICSMIFNKV------AVTLGDVWILLAALKVLSLLWLLYYTVGT--
Human OTOP3 DVGAEERAAATRPRQKSWLVRHFSLLLRRDRQAQKAGQLFSGLLALNVVFLGGAFICSMIFNKV------AVTLGDVWILLATLKVLSLLWLLYYVAST--
Dog Otop3 DVGAEKTGAPASPHQKSWLVRHFSLLLRRDRQAQKAGQLFSGLLALNVVFLGGAFICSMIFNNV------AITLGDVWILLAALKVLSLLWLLYYATCT--
Opossum Otop3 KEDEKELGGPGPSRNKSWLVRHFSLLLRRDRQAQKASQLFSGLLALNVVFLGGAFICSMIFNNV------AITLGDVWILLAVLKVLAFLWLLYHVAVT--
Platypus Otop3 DMGAEETGAPAGRRQKSWLLRHFSLLLRRDRQAQKAGQLFSGLLALNVAFLGGAFICSMIFNNV------AITLGDVWILLATLKVLSLLWLLYYVAGT--
Chicken Otop3 --------------EKSWLYRHCSL-QQRDRQAQKSGQLFSGLLALNVVFLGSAFISSMIFNHV------AITLADVWILLSILKVLCLCWIIYYLLGT--
X. tropicalis Otop3 ----MDLEDPRVKYEHSWLHRHCMSPTTHHRRAQKTGRLFSGLIALNVMVLGAAVVSSVILSNG------IVPEMHIQIFLTVFMLFSSVWALYHLLYV--
Stickleback Otop3 DSSRATPRGVEPVQDSDQRIQDLGLEPGQ-VWAPSGRRLISGLLGLNVVLLGAALVAGQAFNPE------ALKHQEPQAFLLLLMGVSLIWMLWYLLWA--
Fugu Otop3 --------------------------------------LISGLLGLNVVLLGVALITGQLFNSG------GQQNQEARVFLLLLMGVSVAWMLWYLLWA--
D. melanogaster OTOPLa SHTAGSTDSAEKNNAANKEMELKNVMP-QPLQRTSLFIVTSLVYAILLIVVCIAYVISDVTTHR----LPVLYYETFFTYLYGVSILFLLYVFCFLLQESSCCNGGNGGSKPKPQPKEKKSKKA----------------KNADPADSKDAKGSKDSGKAAKG
A. gambiae OTOPLa GHGLGSGAPAPKNNAANKEMELKNVMP-KPLQRTSLFIVTSLVYAMLLIVVCVAYVISDVTTHR----LPVIYYEGFFTYLYGASILFLLYVFCFLLQESSCCNG-----KPKP-PKEKKPKKEKKS------------KKNADVEAGKEGKDGKEA-----G
A. mellifera. OTOPLa NSSPNAQDNAEKNNAANKEMELKNVRS-TPAKKTSLFIIMSFIYAKLLVVVCIAYVISEVVTHK----LPLYYYEGFFTYLYGVSILFLLYVFCFLLQESACCSRGS--DTPPPPPRPPKPPKEP---------------KEKNKKKDKKDKEQKSS----KS
T. castaneum OTOPLa AHG-GSDSAAEKNNAANKEMELKKVQP-KPDKRTSLFIIMSFVYAKLLVVVCIAYVISDVVTHN----IPLYYYEGFFTYLYGMSILFLLYVFCFLLQESTCCSEGEKKEKVKKPPKEKTKEKE----------------KEKKKEEEKKKKEEKERLKKEKK
D. melanogaster OTOPLb SARNGSTAFFDNGAKSFQAKQQKDKNR--RTGNDAISSALSATYCKLLVLLGVCLPITEVISDQ----IPTYVYQGFYVYLYVGSILFVIFLYISAFR---NRSLFNALK----DYH
D. pseudoobscura OTOPLb SARNGSTAFFDNGAKSYQAKQQKEKNR--RTGNDAISSALSATYCKLLVLLGVCLPITEVISEQ----IPTYVYQGFYVYLYVGSILFVIFLYISAFR---NRSLFNALK----DFH
A. gambiae OTOPLb1 -----------SGPLSEQALESRRKNR--RFGDDALSTALSALYAKIVVILGIALPVTEILSSQ----IPANVYQGFYLFLYTVSIVFVIFVYASTMR---RRAVLTLIK----SYH
A. aegypti OTOPLb1 SKASLSQIPHGSGAHSELAMESRRKNR--RIGDDALSTALSALYAKVIVILGIALPVTEILSSQ----IPANVYQGFYLYLYTVSILFVIFVYASNMR---RRAVMTLIK----TYH
T. castaneum OTOPLb FHNQSDRSISEKDATDHKEEFLKKQNR--RMGDDALTVILSALYAKLLVVLGMAFPITEIISKD----VRPFFYQGFYLYLYLGSITFVAYMYATFVR---EKAVNNIIK----SYH
A. gambiae OTOPLb2 -----------SNKTDELSREHRKQQRDSILARNQLGLALSALYAKLLIVMGITLPLTELVARE----APPNFHQPFYLYLYGVSILFCSFLFVARWR---RAAQLEFLSQCPAGAD
A. aegypti OTOPLb2 -----------SLPPA--VMDMRNKNR--AVAEDHFSTAISALYAKLLIVLGIAFPITDVVSES----APHHFYRGFYLYLYTLSFVFAIFLYVVQFR---HNSKMNYI---LGQYS
D. melanogaster OTOPLc ---MGIAFPMAEVISTY----IPPSFYEVYYLYLYIGSMIFLLFMYATLIW-------------GRPKLP--VPIASP--------------------SKSATKASGTDSMDESDT
D. pseudoobscura OTOPLc ---MGVAFPMAEVISTY----IPPSFYEVYYLYLYIGSMIFLLFMYATLLW-------------GRPKLP--TPMATS--------------------PNKTTKTSTSDSMDESDT
A. gambiae OTOPLc --------------------------------GEALATTLSALYGKLLVVMGIAFPMAEVISTY----IPPSFYEGFYLYLYIGSMVFLLFMYTTLLW-------------GKPKMPPVSPIKKQTL------------ASTKKVMHRTSTTCSTDSGEQSDS
A. aegypti OTOPLc -----------------------------MARGEALATTLSALYGKLLVVMGIAFPMAEVISTY----IPPSFYEGFYLYLYIGSMVFLLFMYTTLLW-------------GRPKTPSVSPVKKQ--------------PTKAKIIHRASTTCSTDSGERSDS
A. mellifera. OTOPLc SAVCYPAPTFLDVSEDRK---------WKKLGCDALASTFSALYGKLLVVMGIAFPMAEVISTY----IPPSFYEGFYLYLYFGSMIFLVYMYATLLRD------------GKPKSKK---------------------------RKDVCDLDSSRSSSGAGG
T. castaneum OTOPLc PLAVAPLGYHQQFPVMRRLSRAEKQIEWRQLGADALATTLSALYGKLLVVMGTAFPMAEVISTY----IPPSFYEAYYLYLYIGSMFFLLYMYTVLLRD-------------KRKRKR------------------------------VAEANNNEESDNDTG
C. elegans OTOPLd1 -MESTSTSVAMSTSDGFSSHDDISQWTKNTDAKNFCVNLMTAIYTLILIMVAFVIEISPTWRSE----KMWLEYSIFCVLMYAVAVAYFLYLYLFVLYP---NVINSCIYYLFKKA-----
C. briggsae OTOPLd KVSTASTSYSISTDD-YISHDNEPQWTRNSTAKSFCINMLCSIYALILTMVAFVIEISPTWRSE----DMWLPYSIFCVLMYAVAVSYFIYLYLFVIYP---NIINSCIYLLFRKGWL--V
C. elegans OTOPLd2 KTQNSTLITTPLAMTSDNNHENMKRWTKNPEARIFCINTLSALYALILTMIAFVIEISPTWRSQ----EMWLEYSIFGVLMYLFAILYFTYFYLFVIYP---NIINSIIYFLHRRGRI--A
C. elegans OTOPLe SIESTSSDVTVLTDYSSTIPENLPAWTKNTKAQSFGLKSLTALYTLILTIVAFIIEISPTWQSE----SMNIEYTIFCVLMYSIAILYFIYLYAYILYP---IVFNTILLYLERMRVLNVH
C. briggsae OTOPLe SIESTSSDVTVLSDYSSTLPENLPTWTKNQKAQSFGLKSLTALYTLILTIVAFIVEISPTWQSD----SMHIEYTIFCVLMYSIAILYFIYLYTVILYP---IVFNTILLYLERIHVLSVH
C. elegans OTOPLf LDHV-TVPNALPSPTES--LLEHPQWIRNPKALRFLVGLLTSMYVLLLTIVSCLISISSAWSSP----DLWLAETIFSLLMYLIAILFFMYTYLMIIYP---KPINMLIMILNRKT-SLVP
C. briggsae OTOPLf PGHLPSTPLASPTLSEEPLLDQHPEWIQNPKATRFLVGLLTSMYVLLLTIVSCLISLSSAWQSP----DLWLAETIFSLLMYGIAILFFIYTYFMVIYP---KPINALIMFLNKKRPSWVP
C. elegans OTOPLg MEKGGSDSDSLPSSPTHHHELYRRPWIHEPRATNFFVRLITSLYALILTIISLVVEVSPTWRT-----DMWLAETIFYISMYGVGILFFAYCYIFIIYP---GPYNQLISVLRKYKIIK--
C. briggsae OTOPLg MEKGGSDSDSLPSSPTHHHEIYRRPWIHEPRATNFFVRLITSLYALIITIISLVVEVSPTWRT-----DMWQAETMFYIAMYGVGILFFAYCYVFIIYP---GPYNYLISILRRYKIIK--
C. elegans OTOPLh R---------SLFQSAMMRLLSVAENA-GPSARTSLLTACTVFYALFLTIFSLVLELAHLLNDEE-SRKLNKKDIIFGLYMYGGSLLFFFYMYIVLLLNPRWYSTMEYLKKKNRGTLEKCSPKKCSTNSRQKINIRIPKINNFQKLFGICRTSPKVAPSSDSL
C. briggsae OTOPLh RGGGRRGRSCSLLQTIMLRLLSVDENSLGPSARTSLLTACTVFYALFLTIFSLVLELAHLLNDEE-SRKLNKKDIIFGLYMYGGSLVFFFYMYIVLLLNPRWYSTMDYLG----------------------------------KMFGVCLTRPKVSPSSDSL
C. elegans OTOPLi TSSNCSTIATPNSKVRFRYHEKR-YWLSDAWSRAYVFRLFTCFYAIFIVIAGIVIELSNIIAAEENTGKISIKDLVLGTWLLGGSILFIAYCSYLVHD---MHFTIKRQRISLRN
C. briggsae OTOPLi S-FDASTVGTPNSKIRFRYTDKKEFWLSNAGSRAYVFRLFTCFYAIFIVIAGIVIELSNIIATTENTDKISIKDLVLGTWLLGGSILFIAYCSYLVHD---MHFTIKRRRQNFRN

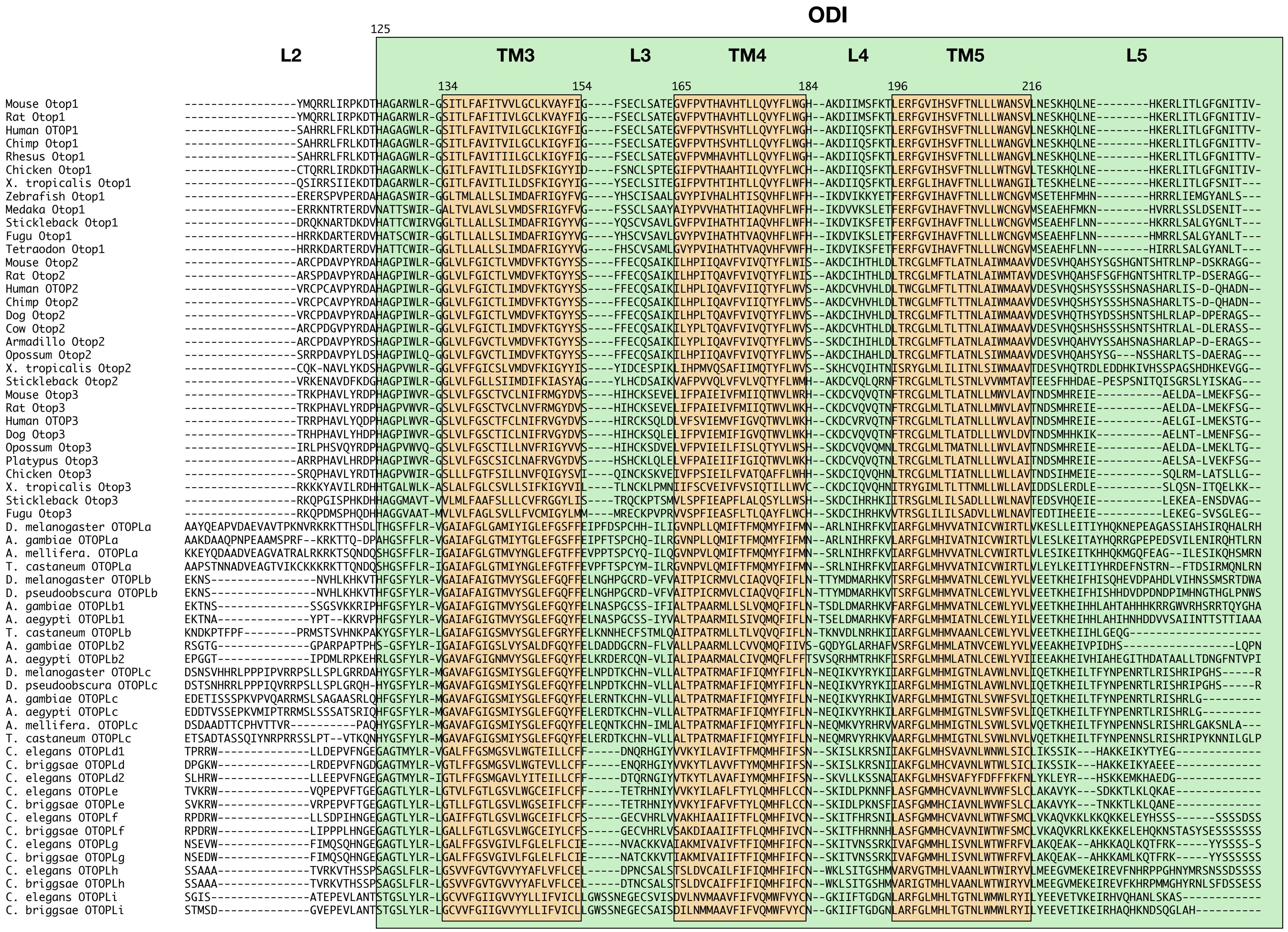
ODI
125
L2
TM3
L3
TM4
L4
TM5
L5
134
154
165
184
196
216
Mouse Otop1
Rat Otop1
Human OTOP1
Chimp Otop1
Rhesus Otop1
Chicken Otop1
X. tropicalis Otop1
Zebrafish Otop1
Medaka Otop1
Stickleback Otop1
Fugu Otop1
Tetraodon Otop1
Mouse Otop2
Rat Otop2
Human OTOP2
Chimp Otop2
Dog Otop2
Cow Otop2
Armadillo Otop2
Opossum Otop2
X. tropicalis Otop2
Stickleback Otop2
Mouse Otop3
Rat Otop3
Human OTOP3
Dog Otop3
Opossum Otop3
Platypus Otop3
Chicken Otop3
X. tropicalis Otop3
Stickleback Otop3
Fugu Otop3
D. melanogaster OTOPLa
A. gambiae OTOPLa
A. mellifera. OTOPLa
T. castaneum OTOPLa
D. melanogaster OTOPLb
D. pseudoobscura OTOPLb
A. gambiae OTOPLb1
A. aegypti OTOPLb1
T. castaneum OTOPLb
A. gambiae OTOPLb2
A. aegypti OTOPLb2
D. melanogaster OTOPLc
D. pseudoobscura OTOPLc
A. gambiae OTOPLc
A. aegypti OTOPLc
A. mellifera. OTOPLc
T. castaneum OTOPLc
C. elegans OTOPLd1
C. briggsae OTOPLd
C. elegans OTOPLd2
C. elegans OTOPLe
C. briggsae OTOPLe
C. elegans OTOPLf
C. briggsae OTOPLf
C. elegans OTOPLg
C. briggsae OTOPLg
C. elegans OTOPLh
C. briggsae OTOPLh
C. elegans OTOPLi
C. briggsae OTOPLi

ODI

L5

```
Mouse Otop1               -------------------------------------------------------------------------------------------------------------------------------------------------------------------
Rat Otop1                 -------------------------------------------------------------------------------------------------------------------------------------------------------------------
Human OTOP1               -------------------------------------------------------------------------------------------------------------------------------------------------------------------
Chimp Otop1               -------------------------------------------------------------------------------------------------------------------------------------------------------------------
Rhesus Otop1              -------------------------------------------------------------------------------------------------------------------------------------------------------------------
Chicken Otop1             -------------------------------------------------------------------------------------------------------------------------------------------------------------------
X. tropicalis Otop1       -------------------------------------------------------------------------------------------------------------------------------------------------------------------
Zebrafish Otop1           -------------------------------------------------------------------------------------------------------------------------------------------------------------------
Medaka Otop1              -------------------------------------------------------------------------------------------------------------------------------------------------------------------
Stickleback Otop1         -------------------------------------------------------------------------------------------------------------------------------------------------------------------
Fugu Otop1                -------------------------------------------------------------------------------------------------------------------------------------------------------------------
Tetraodon Otop1           -------------------------------------------------------------------------------------------------------------------------------------------------------------------
Mouse Otop2               -------------------------------------------------------------------------------------------------------------------------------------------------------------------
Rat Otop2                 -------------------------------------------------------------------------------------------------------------------------------------------------------------------
Human OTOP2               -------------------------------------------------------------------------------------------------------------------------------------------------------------------
Chimp Otop2               -------------------------------------------------------------------------------------------------------------------------------------------------------------------
Dog Otop2                 -------------------------------------------------------------------------------------------------------------------------------------------------------------------
Cow Otop2                 -------------------------------------------------------------------------------------------------------------------------------------------------------------------
Armadillo Otop2           -------------------------------------------------------------------------------------------------------------------------------------------------------------------
Opossum Otop2             -------------------------------------------------------------------------------------------------------------------------------------------------------------------
X. tropicalis Otop2       -------------------------------------------------------------------------------------------------------------------------------------------------------------------
Stickleback Otop2         -------------------------------------------------------------------------------------------------------------------------------------------------------------------
Mouse Otop3               -------------------------------------------------------------------------------------------------------------------------------------------------------------------
Rat Otop3                 -------------------------------------------------------------------------------------------------------------------------------------------------------------------
Human OTOP3               -------------------------------------------------------------------------------------------------------------------------------------------------------------------
Dog Otop3                 -------------------------------------------------------------------------------------------------------------------------------------------------------------------
Opossum Otop3             -------------------------------------------------------------------------------------------------------------------------------------------------------------------
Platypus Otop3            -------------------------------------------------------------------------------------------------------------------------------------------------------------------
Chicken Otop3             -------------------------------------------------------------------------------------------------------------------------------------------------------------------
X. tropicalis Otop3       -------------------------------------------------------------------------------------------------------------------------------------------------------------------
Stickleback Otop3         -------------------------------------------------------------------------------------------------------------------------------------------------------------------
Fugu Otop3                -------------------------------------------------------------------------------------------------------------------------------------------------------------------
D. melanogaster OTOPLa    AGTVLRTH---AGPNSEFEVLD-----GEDILPKDVYKSDNVLSKLVRNTVDGISKSLGMGGDQALDSSTTSSSTTTTTRAPFTTPNYQWHSTTMARKLKKFITSATTAATTAAGSSSTASSTTTISPTISSTTIPSTTISSTTISSSTTFSPFSPSTTTTTT
A. gambiae OTOPLa         AGMVMGMELAPGGTDTEWEPISVNLNAQEDLLSQDA---SSVLSKIVQGTAHT-------------------------------------------------ITEAATTLVSAATLTSTAAPTTTSSSTSTSTS-TTTTTTTTTASPTTTPSWIEPSTGASST
A. mellifera. OTOPLa      AGAILGTAPRDVALLREAP-----------------------LTATRSSTVPT-------------------------------------------------SVASTIAASFGRMVKTTARAIARAVTTPTSTTTTGWNPPTSMTTSTSTPQPSLTTTTASTL
T. castaneum OTOPLa       ANTVLGSH----------------------VAVPDI------------------------------------------------------------------ITTTAAAVTTAPVLTSTLKNLARVTTSTAATTLRGFTTLPTATSARLAEEPLNAINNGT--
D. melanogaster OTOPLb    AVNESLHQHHHH----HALNNTLVANVSS--------------------------------------------------------------------------------------------------------------------------------------
D. pseudoobscura OTOPLb   AMNESLNHHKAHLTVKDPLNHTILANVSS--------------------------------------------------------------------------------------------------------------------------------------
A. gambiae OTOPLb1        RTDHHHLHHDRGRRLVRGAAAALNH------------------------------------------------------------------------------------------------------------------------------------------
A. aegypti OTOPLb1        TSTIDSSEEDYSFQL----DAAVQN------------------------------------------------------------------------------------------------------------------------------------------
T. castaneum OTOPLb       -------------------------------------------------------------------------------------------------------------------------------------------------------------------
A. gambiae OTOPLb2        RTDHRHHHQDDPAQ-----------------------------------------------------------------------------------------------------------------------------------------------------
A. aegypti OTOPLb2        TLNATHHHLTKR-------------------------------------------------------------------------------------------------------------------------------------------------------
D. melanogaster OTOPLc    GHAII-QHDPTAHLR----------------------------------------------------------------------------------------------------------------------------------------------------
D. pseudoobscura OTOPLc   GHAVIPVHDPTAHLR----------------------------------------------------------------------------------------------------------------------------------------------------
A. gambiae OTOPLc         NKMMPHPVETVAHLR----------------------------------------------------------------------------------------------------------------------------------------------------
A. aegypti OTOPLc         HKMIPHPAETVAHLR----------------------------------------------------------------------------------------------------------------------------------------------------
A. mellifera. OTOPLc      --VHNHYHHGEQHLR----------------------------------------------------------------------------------------------------------------------------------------------------
T. castaneum OTOPLc       AVPMDHPPEAHGHHI----------------------------------------------------------------------------------------------------------------------------------------------------
C. elegans OTOPLd1        -------------------------------------------------------------------------------------------------------------------------------------------------------------------
C. briggsae OTOPLd        -------------------------------------------------------------------------------------------------------------------------------------------------------------------
C. elegans OTOPLd2        -------------------------------------------------------------------------------------------------------------------------------------------------------------------
C. elegans OTOPLe         -------------------------------------------------------------------------------------------------------------------------------------------------------------------
C. briggsae OTOPLe        -------------------------------------------------------------------------------------------------------------------------------------------------------------------
C. elegans OTOPLf         SSSEEILEP----------------------------------------------------------------------------------------------------------------------------------------------------------
C. briggsae OTOPLf        SSSEEL-------------------------------------------------------------------------------------------------------------------------------------------------------------
C. elegans OTOPLg         SSSEEI-------------------------------------------------------------------------------------------------------------------------------------------------------------
C. briggsae OTOPLg        SSSEEI-------------------------------------------------------------------------------------------------------------------------------------------------------------
C. elegans OTOPLh         -------------------------------------------------------------------------------------------------------------------------------------------------------------------
C. briggsae OTOPLh        -------------------------------------------------------------------------------------------------------------------------------------------------------------------
C. elegans OTOPLi         -------------------------------------------------------------------------------------------------------------------------------------------------------------------
C. briggsae OTOPLi        -------------------------------------------------------------------------------------------------------------------------------------------------------------------
```

ODI

L5

```
Mouse Otop1               ----------------------------------------------------------------------------------------------------------------------------------------------LDDHTPQCNCTP-PALCSALS
Rat Otop1                 ----------------------------------------------------------------------------------------------------------------------------------------------LDDHTPQCNCTP-PALCSALS
Human OTOP1               ----------------------------------------------------------------------------------------------------------------------------------------------LDDHTPQCNCTP-PTLCTAIS
Chimp Otop1               ----------------------------------------------------------------------------------------------------------------------------------------------LDDHTPQCNCTP-PTLCTAIS
Rhesus Otop1              ----------------------------------------------------------------------------------------------------------------------------------------------LDDHTPQCNCTP-PTLCTAIS
Chicken Otop1             ----------------------------------------------------------------------------------------------------------------------------------------------LDDHAPQCNCTT-TTLCSIFS
X. tropicalis Otop1       ----------------------------------------------------------------------------------------------------------------------------------------------MAHHTPECNCTS--SVCSIFS
Zebrafish Otop1           ----------------------------------------------------------------------------------------------------------------------------------------------TVDVQPHCNCTT--SVCSMFS
Medaka Otop1              ----------------------------------------------------------------------------------------------------------------------------------------------IVSSEPECNSSTTSSTSSLFS
Stickleback Otop1         ----------------------------------------------------------------------------------------------------------------------------------------------IVHSEPHCNCTT--STCSMFS
Fugu Otop1                ----------------------------------------------------------------------------------------------------------------------------------------------IVHAEPQCNCTT--STCSMFS
Tetraodon Otop1           ----------------------------------------------------------------------------------------------------------------------------------------------IVNEEPECNCTI--STCSMFS
Mouse Otop2               -----------------------------------------------------------------------------------------------------------------------------------------------AAEEDPCLCS--TAICQIFQ
Rat Otop2                 -----------------------------------------------------------------------------------------------------------------------------------------------TAEGDPCPCS--TAICQIFQ
Human OTOP2               -----------------------------------------------------------------------------------------------------------------------------------------------PVGGDSCLCS--TAVCQIFQ
Chimp Otop2               -----------------------------------------------------------------------------------------------------------------------------------------------PVGGDSCLCS--TAVCQIFQ
Dog Otop2                 -----------------------------------------------------------------------------------------------------------------------------------------------SVG-GDCSCN--TAICQIFQ
Cow Otop2                 -----------------------------------------------------------------------------------------------------------------------------------------------AVG-GDCSCD--TAVCQIFQ
Armadillo Otop2           -----------------------------------------------------------------------------------------------------------------------------------------------QPGGDPCSCN--TVICQIFQ
Opossum Otop2             ---------------------------------------------------------------------------------------------------------------------------------------------------EACPCN--TTFCQIFE
X. tropicalis Otop2       ------------------------------------------------------------------------------------------------------------------------------------------------AHEHTCQCS--NHLCHIFQ
Stickleback Otop2         ------------------------------------------------------------------------------------------------------------------------------------------------YGDKACTCS--YTSCSIFK
Mouse Otop3               ------------------------------------------------------------------------------------------------------------------------------------------------NGTNTCMCLN-TTVCEVFR
Rat Otop3                 ------------------------------------------------------------------------------------------------------------------------------------------------NGTNTCMCLN-ATVCEVFR
Human OTOP3               ------------------------------------------------------------------------------------------------------------------------------------------------NETNTCLCLN-ATACEAFR
Dog Otop3                 ------------------------------------------------------------------------------------------------------------------------------------------------NDTNTCLCLN-ATVCEVFQ
Opossum Otop3             ------------------------------------------------------------------------------------------------------------------------------------------------NETNTCLCLN-TTICEVFQ
Platypus Otop3            ------------------------------------------------------------------------------------------------------------------------------------------------NETAACLCLN-ATACEVFR
Chicken Otop3             ------------------------------------------------------------------------------------------------------------------------------------------------NETDSCACPN-TTTCKVFQ
X. tropicalis Otop3       -------------------------------------------------------------------------------------------------------------------------------------------------EISACECPT-HSLCWTFK
Stickleback Otop3         -----------------------------------------------------------------------------------------------------------------------------------------------ILNSTLCRCAA-SSACLAFR
Fugu Otop3                -----------------------------------------------------------------------------------------------------------------------------------------------IADLNLCRCGT-NSSCLAFR
D. melanogaster OTOPLa    TTAAALNLE----------TSGSESP---FGGLQRILSSAAPPSLAPVDGFGSASAATPTSGSGAGSFVDSFLAST--LSPASSTEGSASIMNNLFGQGPMENSFQTYTDLGHEEATGLVSFENLESLDNIYPAAL--SSNIGTLNSTA--CGRIDIMGTIVY
A. gambiae OTOPLa         TTARGL-LDRIRDIVATETTTTYETPANHFGSAQHTLSTSA--------GGVNATAATPSTGS---SFLDSFMDHVNYLQRNSSLDQT------------------------------------YESLDALFPSAFIATSTAVSTNATAISCGRVNIMGTIVQ
A. mellifera. OTOPLa      TT-----------------------------------------------------------------------------------------FLTALTPRTTTEERTTPTTPINQIHQIFDLNNQTNSSEYWSPNFGAYAEALTDEEIASNSCGRVNIMGTIVQ
T. castaneum OTOPLa       -----------------------------------------------------------------------------------------------------------------------------EFMEDIIPQALQALNASNGTDVSPQSCGRVNIMGSIVQ
D. melanogaster OTOPLb    -------------------------------------------------------------------------------------------------------------------------------------VIVNMTITPSPTPAAFSGCSRTTIMGALVQ
D. pseudoobscura OTOPLb   -------------------------------------------------------------------------------------------------------------------------------------IIANVTATPSSTPASFTGCSRTTIMGALVQ
A. gambiae OTOPLb1        ----------------------------------------------------------------------------------------------------------------------------------------TLVRRATGADPEYVECQRTNIMGSLVQ
A. aegypti OTOPLb1        ----------------------------------------------------------------------------------------------------------------------------------------RSKRVASHDAAEYIECQRTNIMGTLVQ
T. castaneum OTOPLb       -------------------------------------------------------------------------------------------------------------------------------------------HNSTNSSLQAKFCQEGQIMGSLVR
A. gambiae OTOPLb2        -------------------------------------------------------------------------------------------------------------------------------------------HRLGWAAGSKEPCRSTHIMSTLVH
A. aegypti OTOPLb2        -------------------------------------------------------------------------------------------------------------------------------------------DTLTAPTLHGFECHRTNIMGSLVQ
D. melanogaster OTOPLc    ------------------------------------------------------------------------------------------------------------------------------------------VPRGLKGPYQIFECRRTNIIGTLVQ
D. pseudoobscura OTOPLc   ------------------------------------------------------------------------------------------------------------------------------------------VARGLKGPYQIFECRRTNIIGTLVQ
A. gambiae OTOPLc         ------------------------------------------------------------------------------------------------------------------------------------------VARGLKGPHNIFECRRSNIIGTLVQ
A. aegypti OTOPLc         ------------------------------------------------------------------------------------------------------------------------------------------VARGLKGPHNIFECRRSNIIGTLVQ
A. mellifera. OTOPLc      ------------------------------------------------------------------------------------------------------------------------------------------LPRGLKGPHHMFECRRTNIMGSLVQ
T. castaneum OTOPLc       ------------------------------------------------------------------------------------------------------------------------------------------VARGLKGPHHLYECRRTNIIGSLVQ
C. elegans OTOPLd1        --------------------------------------------------------------------------------------------------------------------------SDNETKTEILTEM----SYDFTTD-SFGPHDKQLRAIIKLG
C. briggsae OTOPLd        --------------------------------------------------------------------------------------------------------------------------AKNATKTEVFTKW----SYDFTMD-EFGPHDKQLRAIIKLG
C. elegans OTOPLd2        --------------------------------------------------------------------------------------------------------------------------LKNQTATNVLAQW----SYNLTTN-SFGAHDKQLRTIVKMG
C. elegans OTOPLe         -----------------------------------------------------------------------------------------------------------------EKYKKKNMTITEAVAETITTTI--ASIVSSESSSGFRADEKQLRSLYKLG
C. briggsae OTOPLe        -----------------------------------------------------------------------------------------------------------------EKWKKKNMT--DMVTEAITTTV--AYVLEGEG--EFSASEKQLRSLYKLG
C. elegans OTOPLf         -----------------------------------------------------------------------------------------------------------ESSTLLENLIEVVLEKLKPGKGTTYTST--PYPYTNSSNTTS---LLRMQSMVRLG
C. briggsae OTOPLf        -----------------------------------------------------------------------------------------------------------EATTVLEGIVEVVVEKMKPIMMSTNSSS--AKTYSSNNYTTSNHTLLRMQSMARLG
C. elegans OTOPLg         -----------------------------------------------------------------------------------------------------------------------------HELISAVLNS--TLNNTPATKTMEPVA-SRLFALEHFG
C. briggsae OTOPLg        -----------------------------------------------------------------------------------------------------------------------------HELISAVLNS--TINNTPETKTVEPAAVSRLFALEHFG
C. elegans OTOPLh         -------------------------------------------------------------------------------------------------------------------------------AENSMEQFLGDGSHEDEHFEKKIMPGSCQAVECILG
C. briggsae OTOPLh        -------------------------------------------------------------------------------------------------------------------------------QEQSSEMF---SSHEDEHDKK--IVGSCQAVECFLG
C. elegans OTOPLi         ------------------------------------------------------------------------------------------------------------------------------------------------AHHHEVHEEACKGVLCIFN
C. briggsae OTOPLi        ------------------------------------------------------------------------------------------------------------------------------------------------GHYHEELEEICKGVLCVFT
```

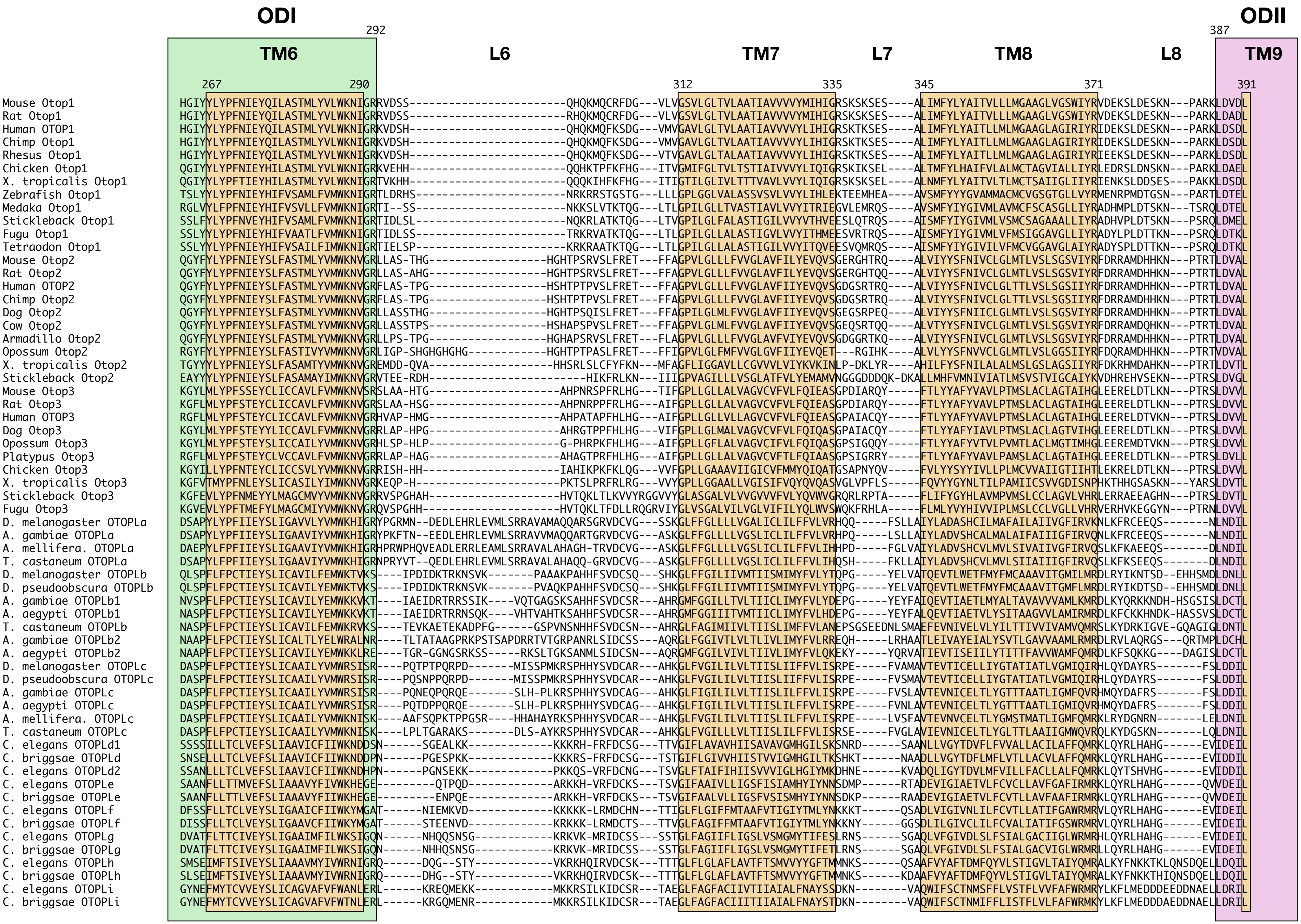
ODI
ODII
292
387
TM6
L6
TM7
L7
TM8
L8
TM9
267
290
312
335
345
371
391
Mouse Otop1
Rat Otop1
Human OTOP1
Chimp Otop1
Rhesus Otop1
Chicken Otop1
X. tropicalis Otop1
Zebrafish Otop1
Medaka Otop1
Stickleback Otop1
Fugu Otop1
Tetraodon Otop1
Mouse Otop2
Rat Otop2
Human OTOP2
Chimp Otop2
Dog Otop2
Cow Otop2
Armadillo Otop2
Opossum Otop2
X. tropicalis Otop2
Stickleback Otop2
Mouse Otop3
Rat Otop3
Human OTOP3
Dog Otop3
Opossum Otop3
Platypus Otop3
Chicken Otop3
X. tropicalis Otop3
Stickleback Otop3
Fugu Otop3
D. melanogaster OTOPLa
A. gambiae OTOPLa
A. mellifera. OTOPLa
T. castaneum OTOPLa
D. melanogaster OTOPLb
D. pseudoobscura OTOPLb
A. gambiae OTOPLb1
A. aegypti OTOPLb1
T. castaneum OTOPLb
A. gambiae OTOPLb2
A. aegypti OTOPLb2
D. melanogaster OTOPLc
D. pseudoobscura OTOPLc
A. gambiae OTOPLc
A. aegypti OTOPLc
A. mellifera. OTOPLc
T. castaneum OTOPLc
C. elegans OTOPLd1
C. briggsae OTOPLd
C. elegans OTOPLd2
C. elegans OTOPLe
C. briggsae OTOPLe
C. elegans OTOPLf
C. briggsae OTOPLf
C. elegans OTOPLg
C. briggsae OTOPLg
C. elegans OTOPLh
C. briggsae OTOPLh
C. elegans OTOPLi
C. briggsae OTOPLi

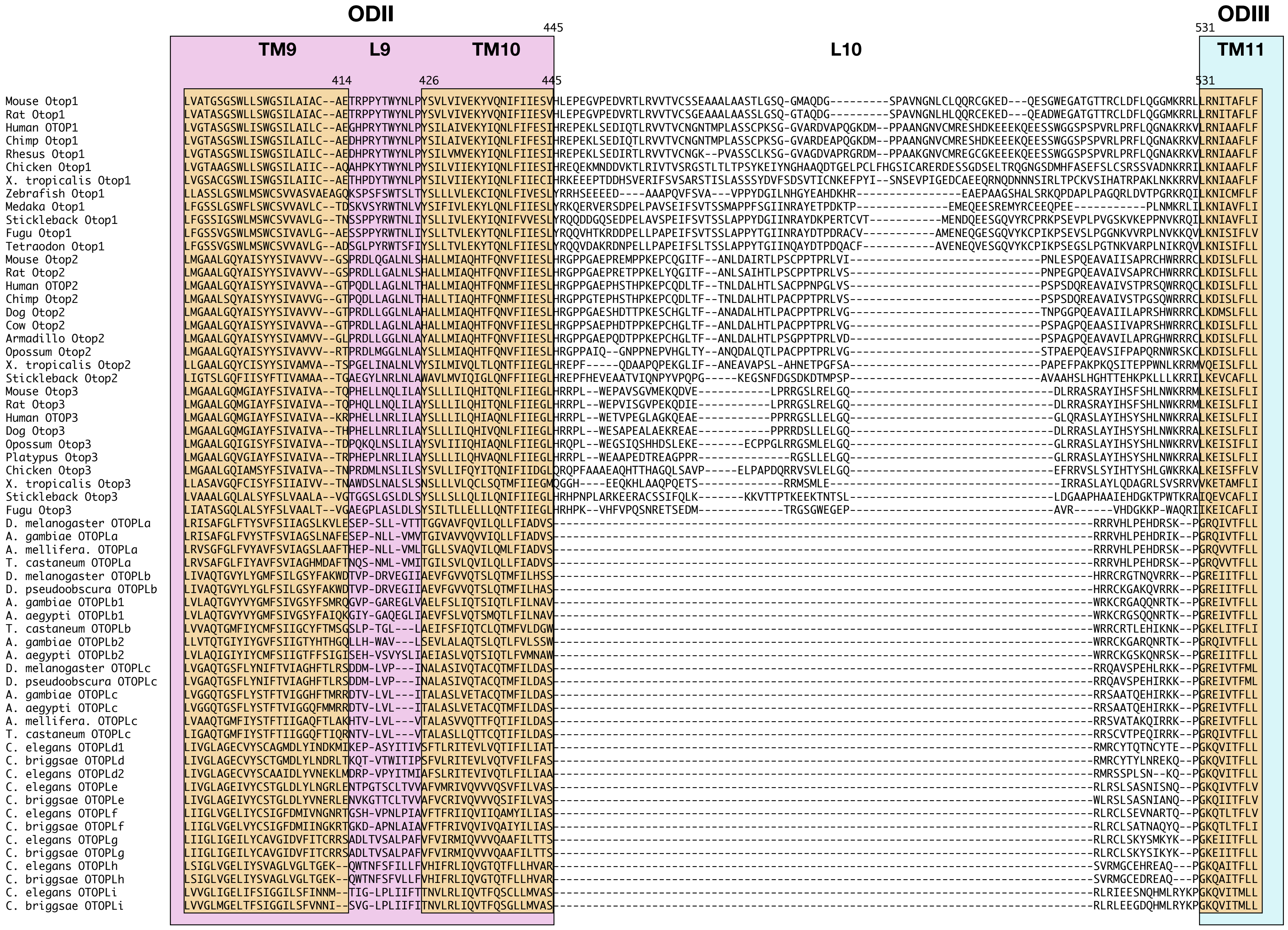
ODII
ODIII
445
531
TM9
L9
TM10
L10
TM11
414
426
445
531
Mouse Otop1
Rat Otop1
Human OTOP1
Chimp Otop1
Rhesus Otop1
Chicken Otop1
X. tropicalis Otop1
Zebrafish Otop1
Medaka Otop1
Stickleback Otop1
Fugu Otop1
Tetraodon Otop1
Mouse Otop2
Rat Otop2
Human OTOP2
Chimp Otop2
Dog Otop2
Cow Otop2
Armadillo Otop2
Opossum Otop2
X. tropicalis Otop2
Stickleback Otop2
Mouse Otop3
Rat Otop3
Human OTOP3
Dog Otop3
Opossum Otop3
Platypus Otop3
Chicken Otop3
X. tropicalis Otop3
Stickleback Otop3
Fugu Otop3
D. melanogaster OTOPLa
A. gambiae OTOPLa
A. mellifera. OTOPLa
T. castaneum OTOPLa
D. melanogaster OTOPLb
D. pseudoobscura OTOPLb
A. gambiae OTOPLb1
A. aegypti OTOPLb1
T. castaneum OTOPLb
A. gambiae OTOPLb2
A. aegypti OTOPLb2
D. melanogaster OTOPLc
D. pseudoobscura OTOPLc
A. gambiae OTOPLc
A. aegypti OTOPLc
A. mellifera. OTOPLc
T. castaneum OTOPLc
C. elegans OTOPLd1
C. briggsae OTOPLd
C. elegans OTOPLd2
C. elegans OTOPLe
C. briggsae OTOPLe
C. elegans OTOPLf
C. briggsae OTOPLf
C. elegans OTOPLg
C. briggsae OTOPLg
C. elegans OTOPLh
C. briggsae OTOPLh
C. elegans OTOPLi
C. briggsae OTOPLi

ODIII

```
                                                                                600
            TM11        L11              TM12                     COOH
                       551             568                587
Mouse Otop1              LCNISLWIPPAFGCRPEYDNGLEEIVFGFEPWIIVVNLAMPFSIFYRMHAAA-----------------ALFEVYCKI-------------------------------------------------------------------------------------
Rat Otop1                LCNISLWIPPAFGCRPEYDNGLEEIVFGFEPWIIVVNLAMPFSIFYRMHAAA-----------------ALFEVYCKI-------------------------------------------------------------------------------------
Human OTOP1              LCNISLWIPPAFGCRPEYDNGLEEIVFGFEPWIIVVNLAMPFSIFYRMHAAA-----------------SLFEVYCKI-------------------------------------------------------------------------------------
Chimp Otop1              LCNISLWIPPAFGCRPEYDNGLEEIVFGFEPWIIVVNLAMPFSIFYRMHAAA-----------------SLFEVYCKI-------------------------------------------------------------------------------------
Rhesus Otop1             LCNISLWIPPAFGCRPEYDNGLEEIVFGFEPWIIVVNLAMPFSIFYRMHAAA-----------------SLFEVYCNI-------------------------------------------------------------------------------------
Chicken Otop1            LCNLSLWIPPAFGCRPEYDNGLEEIVFGFEPWIIVVNLAMPFSIFYRMHSAA-----------------SLFEVNCKT-------------------------------------------------------------------------------------
X. tropicalis Otop1      LCNISLWIPPAFGCRPQYDNGLEEIVFGFEPWITVVNLAMPFSIFYRMHSAS-----------------SLFEVYCKA-------------------------------------------------------------------------------------
Zebrafish Otop1          MCNISLWILPAFGCRPQYDNPLENETFGTSVWTTVLNVAIPLNLFYRMHSVA-----------------SLFEVFRKV-------------------------------------------------------------------------------------
Medaka Otop1             MCNISLWLLPAFGCRPQYDNGLEQDIYGFSAWTTVLNFAIPLNLFYRMHSVA-----------------SLFEVFRRV-------------------------------------------------------------------------------------
Stickleback Otop1        MCNISLWILPAFGCRPQYDNGLEQETFGFSVWTTVLNFAIPLNLFYRMHSVA-----------------SLFEVFRRV-------------------------------------------------------------------------------------
Fugu Otop1               MCNISLWILPAFGCRPQYDNGLEQETFGFSIWTTVLNFAIPLNLFYRMHAVS-----------------SLFEVFCQV-------------------------------------------------------------------------------------
Tetraodon Otop1          MCNICLWILPAFGCRPQYDNGLEEEMFGFSIWTAVLNFAIPLNLFYRMHSVF-----------------SLFDVFCQ--------------------------------------------------------------------------------------
Mouse Otop2              LCNVILWIMPAFGARPHFSNTVEVDFYGYSLWAAIVNICLPFGIFYRMHAVS-----------------SLLEVYVLS-------------------------------------------------------------------------------------
Rat Otop2                LCNVILWIMPAFGARPHFSNTVEVDFYGYSLWAAIVNICLPFGIFYRMHAVS-----------------SLLEVYVLS-------------------------------------------------------------------------------------
Human OTOP2              LCNVILWIMPAFGARPHFSNTVEVDFYGYSLWAVIVNICLPFGIFYRMHAVS-----------------SLLEVYVLS-------------------------------------------------------------------------------------
Chimp Otop2              LCNVILWIMPAFGARPHFSNTVEVDFYGYSLWAVIVNICLPFGIFYRMHAVS-----------------SLLEVYVLS-------------------------------------------------------------------------------------
Dog Otop2                LCNVILWIMPAFGARPHFSNTVEVDFYGYSLWAAIVNICLPFGIFYRMHAVS-----------------SLLEVYVLS-------------------------------------------------------------------------------------
Cow Otop2                LCNIILWIMPAFGARPHFSNTVEVDFYGYSLWAAIVNICLPFGIFYRMHAVS-----------------SLLEVYVMS-------------------------------------------------------------------------------------
Armadillo Otop2          LCNIVLWIMPAFGARPHFRNTVEVDFYGYSLWAAIVNICLPFGIFYRMHAVS-----------------SLLEVYVLS-------------------------------------------------------------------------------------
Opossum Otop2            LCNVILWIMPAFGARPHFNNNLELDFYGYSLWAAIVNICLPFGIFYRMHAVS-----------------SLLEVYVLS-------------------------------------------------------------------------------------
X. tropicalis Otop2      LCNIILWLMPAFGARPQLKNNLERNFYGLYVWTIITNICLPFGIFYRMHAAA-----------------SLLEVFRMS-------------------------------------------------------------------------------------
Stickleback Otop2        LGNIILWIMPAFGARPQFDHDTETEFYKLNMWATIVNIGLPFGIFYRMHSVA-----------------SLFEVFLTS-------------------------------------------------------------------------------------
Mouse Otop3              LCNITLWMMPAFGIHPEFENGLEKDFYGYRTWFTIVNFGLPLGVFYRMHSVG-----------------GLVEVYLGA-------------------------------------------------------------------------------------
Rat Otop3                LCNITLWMMPAFGIHPEFENGLEKDFYGYRTWFTIVNFGLPLGVFYRMHSVG-----------------GLVEVYLGA-------------------------------------------------------------------------------------
Human OTOP3              LCNITLWMMPAFGIHPEFENGLEKDFYGYQIWFAIVNFGLPLGVFYRMHSVG-----------------GLVEVYLGA-------------------------------------------------------------------------------------
Dog Otop3                LCNITLWMMPAFGIHPEFENGLEKDFYGYRTWFTIVNFGLPLGVFYRMHSVG-----------------GLVELYLEA-------------------------------------------------------------------------------------
Opossum Otop3            ICNITMWMMPAFGTHPEFENGLEKSFYGYRTWFTIVNFGLPLGVFYRMHSVG-----------------GLVEVYLGA-------------------------------------------------------------------------------------
Platypus Otop3           LCNI-LWMMPAFGTHPEFENGLEKEFYGYRTWFTIVNTGLPLGVFYRMHSVG-----------------GLVEVYLGA-------------------------------------------------------------------------------------
Chicken Otop3            LCNIILWIMPTFGAHPVFENGLQKSFYGYSTWFAIVNFGLPLSVFYRMHSVG-----------------GLLEVYVSA-------------------------------------------------------------------------------------
X. tropicalis Otop3      LCNIMCWIMGAFGAHPLYMNGLERHFYGSTIWLAILNIGLPLSVFYRMHSVG-----------------SLMEVYIHA-------------------------------------------------------------------------------------
Stickleback Otop3        LSNVMLWVIPAFGVHPQFENGLGKQFFGFSPWFVLVNLGQPLGVFYRMHSVG-----------------ALMELLIAA-------------------------------------------------------------------------------------
Fugu Otop3               LSNIMMWVIPAFGVHPQFEIGFGKQFFGFSAWFALVNLSQPLSVFYRMHSVA-----------------ALMELLL---------------------------------------------------------------------------------------
D. melanogaster OTOPLa   ICNVAMFAIYTFEAQKVFANPVQLEFYGFVPWSIIQRITLPLCIFHRFHSAV-----------------TLAEIWKTTYKARLE-------------------------------------------------------------------------------
A. gambiae OTOPLa        ICNISMFAIYTFEAQKVFANPVQLDFYGFIAWSLIQRVTLPLCIFHRFHSAV-----------------TLAEVWKTTYKARLE-------------------------------------------------------------------------------
A. mellifera. OTOPLa     ICNLTMWVIYTFESQKVMANPVQLDFYGFLAWAIVQRVTLPLCIFHRFHSAV-----------------TFAEIWKTSYKARLD-------------------------------------------------------------------------------
T. castaneum OTOPLa      ICNVTMWIIYTFEMQKVKANPVQLKFYGFLAWAIVQRFTLPLCIFHRFHSAV-----------------TLAEIWKTSYKARLE-------------------------------------------------------------------------------
D. melanogaster OTOPLb   VANIAIWFVNTLIKGRAVFRESHLEFFGVWGWTIITHISMPLAIFYRFHSTI-----------------CLFEVWKITYKAKAH-------------------------------------------------------------------------------
D. pseudoobscura OTOPLb  VANIAIWFVNTLIKGRAVFRESHLEFFGVWGWTIITHISMPLAIFYRFHSTI-----------------CLFEVWKITYKAKAH-------------------------------------------------------------------------------
A. gambiae OTOPLb1       VANMAMWFINTLIKGRASFRPSHLDFFGTWAWTVITHVSMPLAIFYRFHSTI-----------------CLFEVWKSTYKVKINDHH----------------------------------------------------------------------------
A. aegypti OTOPLb1       VANMAMWFINTLVKGRASFRPTHLDFFGTWAWTVITHVSMPLAIFYRFHSTI-----------------CLFEIWKSTYKVKSGEHH----------------------------------------------------------------------------
T. castaneum OTOPLb      VANMAMWTINTLEKNRAEFRPTHLLFFGEWAWTIITHISMPMAIFYRFHSTI-----------------CLFEIWKTAYKVKTQYKTPMFSVISNQ-------------------------------------------------------------------
A. gambiae OTOPLb2       VANLAAWMVNSLVKSNASFRPLVMGFYGAGAWAIIAHISMPLAIFYRFHSTI-----------------CLFEIWKNSYKARYTEG-----------------------------------------------------------------------------
A. aegypti OTOPLb2       ISNMSIWFINTLIKTNASFRPSLMKFYGVWAWTIITHISMPLAIFYRFHSTI-----------------CLFEVWKNTYKVRYEHN-----------------------------------------------------------------------------
D. melanogaster OTOPLc   VVNLAMWAISTLEKSRAESHPIQLNFYGLWAWTIITHVSMPLAIFYRFHSTSIAQQQQFCEDLKTNLSYCYCSTTLAGGELETVEEVDSGESNSAEDAGAGAGSGGSRGSGGGAGAAEAGEAGEEGQQGGDSSCGLKAPIRALSPQSLNTEKAFCPVYVINGE
D. pseudoobscura OTOPLc  VVNLAMWAISTLEKSRAESHPIQLNFYGLWAWTIITHVSMPLAIFYRFHSTVIAQQQQFCEDLKTNLSYCYCSTTLAGGELETVEEVDSGENQEKIEA-------------------EAGKEEVKDALGGDS------GRRALSPQSLNTEKAFCPVYVINGE
A. gambiae OTOPLc        VSNLAMWAINTLEKSRAESHPIQLHFYGLWAWTIITHVSMPLAIFYRFHSTV-----------------CLCEIWKRAYKMKPAYM-----------------------------------------------------------------------------
A. aegypti OTOPLc        VSNLAMWAINTLEKSRAESHPIQLHFYGLWAWTIITHVSMPLAIFYRFHSTV-----------------CLCEIWKRAYKMKPAYM-----------------------------------------------------------------------------
A. mellifera. OTOPLc     VTNLAMWAINTLEKSRAESHPVQLHFYGLWAWTIITHVSMPLAIFYRFHSTV-----------------CLCEVWKRAYKVKPTYM-----------------------------------------------------------------------------
T. castaneum OTOPLc      VTNLAMWAINTLEKSRAESHPIQLHFYGLWAWTIITHVSMPLAIFYRFHSTV-----------------CLCEIWKRAYKIKPTYM-----------------------------------------------------------------------------
C. elegans OTOPLd1       VCNINLFIYHTFETTELNFG---FPIKLPDSYSAMLNIASPIVVFYRFHSSA-----------------CLAEIWKHTYSRKNHNVVTDNTVDP--LVVVKKM------------------------------------------------------------
C. briggsae OTOPLd      VCNINLFIYHTFETMESNFG---FPLKLPDNYSVMLSIASPIVVFYRFHSSA-----------------CLAEIWKHTYSRKHHHEQLIHEPTTE-NTVVKKL------------------------------------------------------------
C. elegans OTOPLd2       ICNINLFIYHTFETTESSFG---FPAKVSEVYTVMLTIASPIVVFYRFHSSA-----------------CLAEIWKNTYLRNGHDANVGPADSNEKLMMIDKL------------------------------------------------------------
C. elegans OTOPLe       VCNINLFIYHTFETIESNFG---FPHKMSSVYSALLNISSPLVVFYRFHSSA-----------------CLAEIWKHAYSKKYNHSHNAHKSLDDLHVV----------------------------------------------------------------
C. briggsae OTOPLe      VCNINLFIYHTFETIESNFG---FPHKMASVYSALLNISSPLVVFYRFHCSA-----------------CLAEIWKHAYSTKHNHSHNHNVHHKPLEDHHIA-------------------------------------------------------------
C. elegans OTOPLf       LINITLFVYHTFEGMKSTYG---FPALMETKYFELLNISSPLVVFYRFHSSA-----------------CLAEIWKHAYSTKHHHHSHQTPINSPTSESQHHV------------------------------------------------------------
C. briggsae OTOPLf      IINITLFVYHVFEGMKNMYG---FPSIMENKYFELLNISSPLVVFYRFHSSA-----------------CLAEIWKHTYSTKHNHHHHNVQSPISSPINSPMSDASHHHQQLV--------------------------------------------------
C. elegans OTOPLg       VSNVTLFVFHTFEGMKSSFG---FSSKAATQYNYIIYAVGPLLVFYRFHSSA-----------------CLAEIWKHTYSTKSNEYDHEH-MSLSDSNLTAITPISDIKEIPS-PQLLKH-------------------------------------------
C. briggsae OTOPLg      VSNVTLFVFHTFEGMKSSFG---FSSKAATQYNYIIYAVGPLLVFYRFHSSA-----------------CLAEIWKHTYSTKSSEYDHEHIMSLSDSNLTAITPISDSGKIEDVPSLADH-------------------------------------------
C. elegans OTOPLh       TANLAIFFMNLFESEKAGVSEMIIEYYGKRSWVFLVRSFSPLTIFYRFHSSV-----------------CFAEIWKNVYAAKTHLVHNSATSTATPINI----------------------------------------------------------------
C. briggsae OTOPLh      TANLAIFFMNLFESEKAGVSEIIIEYYGKRSWVFLVRSFSPLTIFYRFHSSV-----------------CFAEIWKNVYAAKTHPVHNSATSTATPINI----------------------------------------------------------------
C. elegans OTOPLi       MMNCAQFMMNVFEAQKAGINDEMIHMYGSYYWAIVVRGCSPLTIFYRFHSSA-----------------CFAEIWKKTFRPPKKPKEIENEHTPL--------------------------------------------------------------------
C. briggsae OTOPLi      MMNCAQFLMNVFEAQKAGINDEMISMYGSYYWAIIVRGCSPLTIFYRFHSSA-----------------CFAEVWKKTFRPPKKPKENEEERTQL--------------------------------------------------------------------
```
